# Deep Sequencing Analysis of Individual HIV-1 Proviruses Reveals Frequent Asymmetric Long Terminal Repeats

**DOI:** 10.1128/jvi.00122-22

**Published:** 2022-06-08

**Authors:** Kevin W. Joseph, Elias K. Halvas, Leah D. Brandt, Sean C. Patro, Jason W. Rausch, Abha Chopra, Simon Mallal, Mary F. Kearney, John M. Coffin, John W. Mellors

**Affiliations:** a University of Pittsburgh, Department of Medicine, Division of Infectious Diseases, Pittsburgh, Pennsylvania, USA; b HIV Dynamics and Replication Program, National Cancer Institute, Frederick, Maryland, USA; c Basic Research Laboratory, Center for Cancer Research, National Cancer Institute, Frederick, Maryland, USA; d Institute for Immunology & Infectious Diseases, Murdoch University, Murdoch, Western Australia, Australia; e University of Vanderbilt, Department of Medicine, Nashville, Tennessee, USA; f Tufts University, Boston, Massachusetts, USA; Ulm University Medical Center

**Keywords:** HIV-1, single genome, LTR, integration sites, sequencing, proviral structures

## Abstract

Effective strategies to eliminate human immunodeficiency virus type 1 (HIV-1) reservoirs are likely to require more thorough characterizations of proviruses that persist on antiretroviral therapy (ART). The rarity of infected CD4^+^ T-cells and related technical challenges have limited the characterization of integrated proviruses. Current approaches using next-generation sequencing can be inefficient and limited sequencing depth can make it difficult to link proviral sequences to their respective integration sites. Here, we report on an efficient method by which HIV-1 proviruses and their sites of integration are amplified and sequenced. Across five HIV-1-positive individuals on clinically effective ART, a median of 41.2% (*n* = 88 of 209) of amplifications yielded near-full-length proviruses and their 5′-host-virus junctions containing a median of 430 bp (range, 18 to 1,363 bp) of flanking host sequence. Unexpectedly, 29.5% (*n* = 26 of 88) of the sequenced proviruses had structural asymmetries between the 5′ and 3′ long terminal repeats (LTRs), commonly in the form of major 3′ deletions. Sequence-intact proviruses were detected in 3 of 5 donors, and infected CD4^+^ T-cell clones were detected in 4 of 5 donors. The accuracy of the method was validated by amplifying and sequencing full-length proviruses and flanking host sequences directly from peripheral blood mononuclear cell DNA. The individual proviral sequencing assay (IPSA) described here can provide an accurate, in-depth, and longitudinal characterization of HIV-1 proviruses that persist on ART, which is important for targeting proviruses for elimination and assessing the impact of interventions designed to eradicate HIV-1.

**IMPORTANCE** The integration of human immunodeficiency virus type 1 (HIV-1) into chromosomal DNA establishes the long-term persistence of HIV-1 as proviruses despite effective antiretroviral therapy (ART). Characterizing proviruses is difficult because of their rarity in individuals on long-term suppressive ART, their highly polymorphic sequences and genetic structures, and the need for efficient amplification and sequencing of the provirus and its integration site. Here, we describe a novel, integrated, two-step method (individual proviral sequencing assay [IPSA]) that amplifies the host-virus junction and the full-length provirus except for the last 69 bp of the 3′ long terminal repeat (LTR). Using this method, we identified the integration sites of proviruses, including those that are sequence intact and replication competent or defective. Importantly, this new method identified previously unreported asymmetries between LTRs that have implications for how proviruses are detected and quantified. The IPSA method reported is unaffected by LTR asymmetries, permitting a more accurate and comprehensive characterization of the proviral landscape.

## INTRODUCTION

Human immunodeficiency virus type 1 (HIV-1) is a pathogenic lentivirus that was estimated in 2020 to have infected 37,700,000 people globally and was responsible for approximately 680,000 deaths that same year (1). Vaccination and curative interventions remain an unmet need due, at least in part, to the highly adaptable and polymorphic nature of HIV-1. Factors contributing to HIV-1 diversity include a high replicative capacity, an error-prone viral polymerase (reverse transcriptase [RT]), and errors incorporated into nascent viral genomes by host RNA polymerase II (2–5). Recombination is another important mechanism of HIV-1 diversity, as HIV-1 RT, a low-processivity enzyme that lacks a proofreading domain, is prone to frequent template switching during reverse transcription (6). Collectively, these factors contribute to the accumulation of nucleotide substitutions, small indels, and large deletions in the double-stranded HIV-1 genomic DNA (dsDNA) intermediates formed by reverse transcription (2). The virally encoded integrase enzyme catalyzes the insertion of these intermediates into the host cell genome at various locations (7), where they can persist indefinitely, even in the face of complete suppression of viral replication by antiretroviral therapy (ART) (8–11). However, the proviral landscape does not remain static, even in the absence of virus replication, since T-cells, including those infected with HIV, undergo homeostatic or antigen-induced proliferation (12). The resultant clonal populations of infected T-cells harbor proviruses having identical sequences and sites of integration, which may be used to track these populations longitudinally or among T-cell subtypes.

The majority (>90%) of HIV-1 proviruses in individuals on long-term suppressive ART are defective containing lethal point mutations, indels, rearrangements, and/or APOBEC3G-induced hypermutations; however, some defective proviruses may express mRNA and viral proteins with potential for clinical relevance (2, 4, 13, 14). A small fraction of proviruses is intact and able to produce replication-competent virus that can lead to rebound viremia if suppressive ART is stopped. Intact proviruses in persons on suppressive ART can persist in long-lived CD4^+^ T-cells, and their progeny form cell clones. Although most intact proviruses are latent, some are expressed and produce low-level viremia that is not suppressible with current ART regimens (15–18).

A deeper characterization of the HIV reservoir requires the identification of full-length proviral genomes and their respective integration site (IS) (9–11). An in-depth assessment of proviruses and their genomic locations could explain why some proviruses are expressed, whereas others are latent or noninducible, as the integration site and proviral structure may play critical roles in expression (19). One of the significant barriers to comprehensively analyzing the proviral landscape in persons on ART is the inefficiency and limited throughput of methods available to selectively amplify, sequence, and phenotype full-length proviruses, including their integration locations as well as their orientations relative to the host chromosome (20).

Methods to sequence individual HIV-1 proviruses and their integration sites are generally inefficient because of the difficulty in enriching for polymorphic and rare proviral targets (20). On average, only 1 in 1,000 CD4^+^ T-cells harbors a provirus *in vivo* (4); thus, obtaining rare proviral sequences and their integration sites has generally required increasing single proviral template copy numbers through multiple-displacement amplification (MDA). MDA replicates copies of the proviral template that can then be utilized in downstream PCR analyses, whereas a single template would be consumed in one PCR without pre-amplification by MDA (15, 21, 22). Following MDA, a near-full-length (NFL) HIV-1 amplicon or multiple smaller subgenomic regions are PCR amplified from the MDA product (10, 15, 22). With an aliquot from the same MDA product, the integration site can be amplified using a variety of techniques: ligation-mediated PCR (LM-PCR), integration site loop amplification (ISLA), or nonrestrictive linear amplification-mediated PCR (nrLAM-PCR) (10, 11, 22, 23).

The development of methods to amplify and sequence across HIV-1 host-virus junctions has helped advance our understanding of the proviral landscape, including the discovery of crucial clonally expanded cells harboring intact proviruses. However, the current methods have limitations. For instance, LM-PCR involves the ligation of a sequence-specific linker to fragmented genomic DNA (gDNA), followed by the amplification of the host-virus junction using long terminal repeat (LTR)- and linker-specific primers and next-generation sequencing (NGS) of LM-PCR amplicons (10, 21). LM-PCR-based methods are often limited by off-target priming by the linker or HIV-1 primers, which lowers specificity, efficiency, and throughput. Alternatively, the ISLA method relies on random decamers tagged with a U5 sequence that needs to loop back onto the LTR, followed by two or three rounds of PCR using only HIV-specific primers (11, 21). ISLA is limited by its reliance on the loop-back mechanism, which restricts the amount of flanking host sequence that can be amplified, and requires the use of two primers that bind in the HIV-1 R region that typically amplify only a fraction of the HIV-1 LTR (11). nrLAM-PCR exploits linear amplification across the host-virus junction, followed by single-stranded linker ligation that allows downstream LM-PCR. Single-strand-based ligations are generally inefficient and yield only approximately 20 nucleotides (nt) of provirus at the host-virus junction (23). To enrich proviral templates, most methods also rely on commercial kits for MDA or general whole-genome workflows, which can generate substantial template-independent DNA amplification (TIDA) (24). TIDA is produced by the self-annealing and extension of random primers (often hexamers) during the long isothermal reaction (24). TIDA can increase the off-target-to-target DNA ratio, reduce the amplification efficiency across host-virus junctions, and reduce the specificity of sequencing reads, requiring much greater sequencing depths than with specific amplicon sequencing (24). The low specificity of the sequencing reads reduces the throughput and increases sequencing costs. Finally, existing integration site analysis methods amplify and sequence only a small portion of the flanking host integration sequence and HIV-1 LTR, which can lead to ambiguities in mapping the host sequence to a specific integration site and provide minimal to no sequence overlap with typical near-full-length proviral PCR assays (11, 22, 23, 25).

Here, we report on the individual proviral sequencing assay (IPSA), a high-throughput workflow for efficiently obtaining more of the sequence of a given provirus (i.e., all but 69 nucleotides of the 3′-LTR U5 region) and the 5′-flanking host sequence (median, 430 nucleotides) than any previously published methodology. We use the IPSA to efficiently characterize 88 proviruses from five individuals infected with HIV-1 subtype B on suppressive ART, four of whom were previously found to harbor “repliclones” (replication-competent proviruses in clonally expanded cells) (15). We identified both intact and defective proviruses, proviruses in clonally expanded cells, and frequently found proviruses with asymmetrical LTR structures, a new discovery. Finally, we validated our methodology using an integration-site-specific PCR screening protocol for which custom combinations of host- and HIV-1-specific primers were designed in accordance with our IPSA findings, a process termed “host-to-full-length provirus-to-host” (HFH) (15), applied to unmodified gDNA extracted from peripheral blood mononuclear cells (PBMC).

## RESULTS

### Sample donors.

All participants (*n* = 5) were on long-term ART (median, 10 years; range, 2 to 19 years), but all exhibited episodes of non-suppressible low-level viremia that was shown previously to be of clonal origin and not from ongoing cycles of replication (15) (Table 1). The median nadir CD4^+^ T-cell count was 133 cells/mm^3^ (range, 10 to 314 cells/mm^3^). At the time of sample collection for the study, the median CD4^+^ T-cell count was 533 cells/mm^3^ (range, 380 to 1,023 cells/mm^3^), and the median HIV-1 DNA copy number was 1,533 copies/10^6^ PBMCs (range, 373 to 2,505 copies).

**TABLE 1:** Characteristics of individuals evaluated by the individual proviral sequencing assay^*a*^

Donor ID	Age (yrs)	Sex	Race^*b*^	Nadir CD4 T-cell count (cells/mm^3^)	No. of yrs on ART^*c*^	Current CD4^+^ T-cell count (cells/mm^3^)	No. of HIV-1 DNA copies/10^6^ PBMC	Current ART regimen^*d*^
R-09	73	Male	C	105	10	380	1,533	TDF/FTC/EFV
C-03	43	Male	C	10	9	416	2,505	DRV/r/ETV/DTG
C-02	62	Male	AA	286	18	1,022	373	TDF/FTC/EFV
F-07	59	Male	AA	314	19	1,023	1,603	ABC/3TC/EFV
K-01	55	Female	AA	133	2	533	1,383	TDF/FTC/DRV/r

Median	59			133	10	533	1,533	

a Data modified from reference 15.

b C, Caucasian; AA, African American.

c Years on antiretroviral therapy (ART) at the time of initial evaluation.

d Antiretroviral drugs were ABC (abacavir), ATV/r (atazanavir/ritonavir), DRV/c (darunavir/cobicistat), DRV/r (darunavir/ritonavir), DTG (dolutegravir), EFV (efavirenz), ETV (etravirine), FTC (emtricitabine), TDF (tenofovir disoproxil fumarate), and 3TC (lamivudine).

### Workflow for individual HIV-1 proviral amplification and sequencing (IPSA).

**FIG 1:**
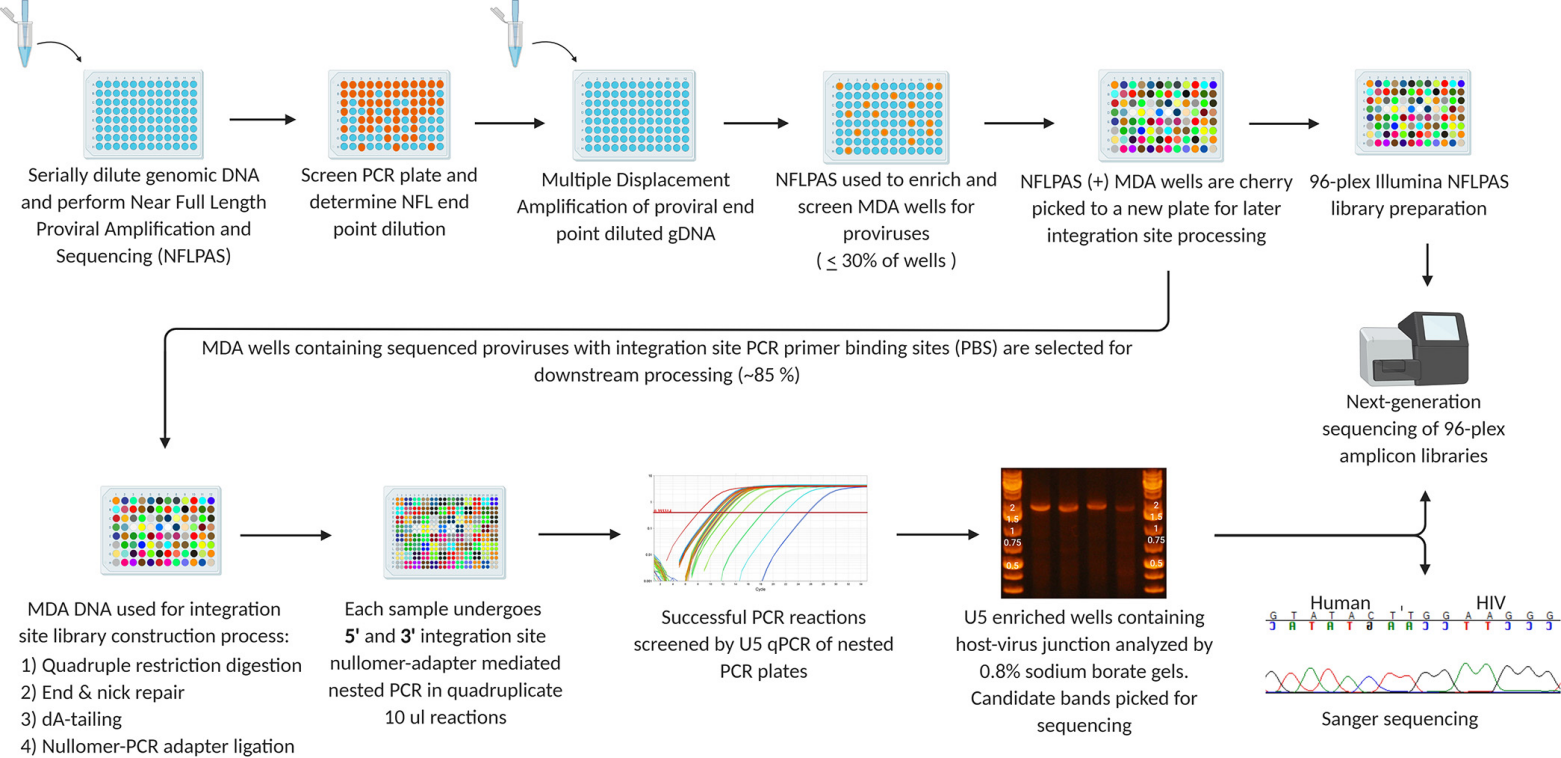
Workflow for amplifying and sequencing individual HIV-1 proviruses and integration sites. gDNA extracted from cells is serially diluted and used for nested near-full-length (NFL) proviral amplification and sequencing (NFLPAS) to determine the proviral endpoint dilution factor. gDNA is then diluted and used in MDA, followed by the screening of MDA reactions for HIV-1 proviruses using NFL proviral amplification and sequencing. Gel red nucleic acid stain is used to identify NFL-positive wells. NFL-positive MDA reactions are then sequenced using the Illumina MiSeq platform, and MDA reactions containing proviruses without integration site PCR primer-binding-site deletions are selected for integration site amplification. A portion of the remaining MDA DNA is restriction digested, end repaired, dA -tailed, nullomer linker ligated, and followed by quadruplicate integration site nested PCR. PCR reactions are screened for U5 enrichment by EvaGreen qPCR, analyzed by 0.8% sodium borate agarose gel electrophoresis, and sequenced with either the Sanger or Illumina MiSeq platforms.

The single proviral endpoint dilution is defined by Poisson distribution such that diluted gDNA from PBMC generates near-full-length (NFL) proviral amplicons in <30% of PCR reactions (Fig. 1). NFL amplicons are variable in size and range from small (<1 kb) to full length (>9 kb). gDNA is diluted and spread across a 96-well plate at the single proviral endpoint, and MDA reactions are performed to uniformly amplify human and viral DNA. Nested PCR is then performed on MDA product fractions with primers designed to amplify proviral sequences from the *gag* leader to 69 bp from the extreme 3′ -terminus of the 3′ LTR. Successful PCR reactions are identified by gel red dye screening and used for library construction and NGS. The corresponding MDA products are then resampled for integration site (IS) determination. The NFL and IS amplicons are sequenced with the Illumina MiSeq platform, their overlapping (49 nt) *gag* leader sequences are aligned for consensus sequence generation, and the overlapping 5′- and 3′-LTR sequences are compared for sequence identity. The workflow utilizes modified MDA conditions (24, 26) that reduce TIDA and improve the sensitivity of downstream proviral PCR by incorporating motif-specific MDA primers (26). Specifically, we designed 10-mer primers (decamers) that are present in the human genome but not in the consensus B HIV-1 genome to favor the synthesis of long MDA amplicons and prevent the synthesis of products containing only part of an HIV-1 provirus. Similarly, we used nullomer sequences (not present in the human genome or HIV-1 consensus B genome) in the design of IPSA adapters to permit the amplification of the 5′ LTR and a portion of *gag* in its entirety while minimizing the likelihood of generating LM-PCR artifacts (27). Collectively, we found that these innovations both improved the specificity, amplification efficiency, and percentage of on-target NGS reads for proviral sequence assembly and allowed us to generate individual chimeric host-viral DNA amplicons of sufficient purity for IS determination by Sanger population sequencing.

### Efficiency metrics for the IPSA.

**FIG 2:**
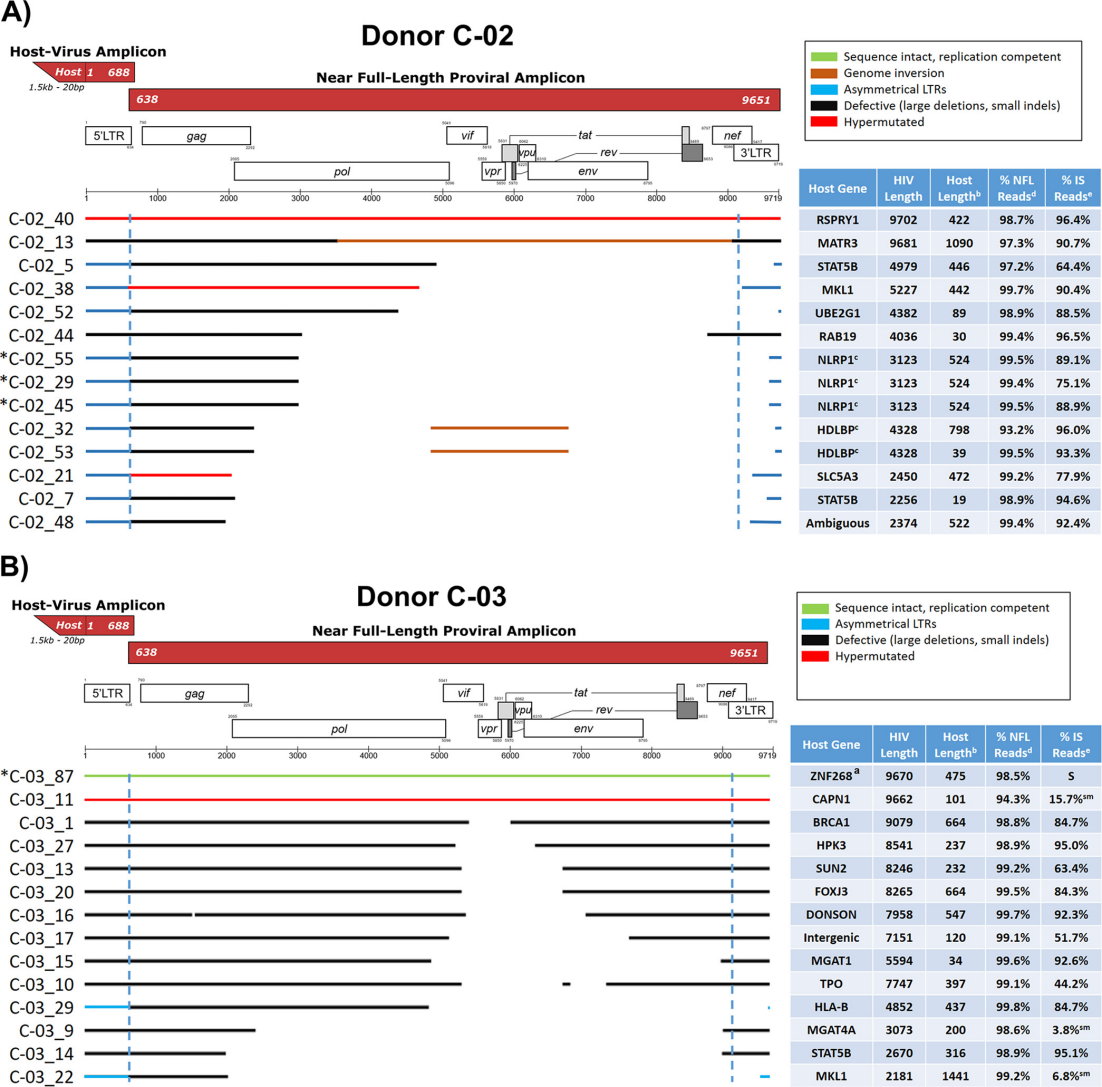

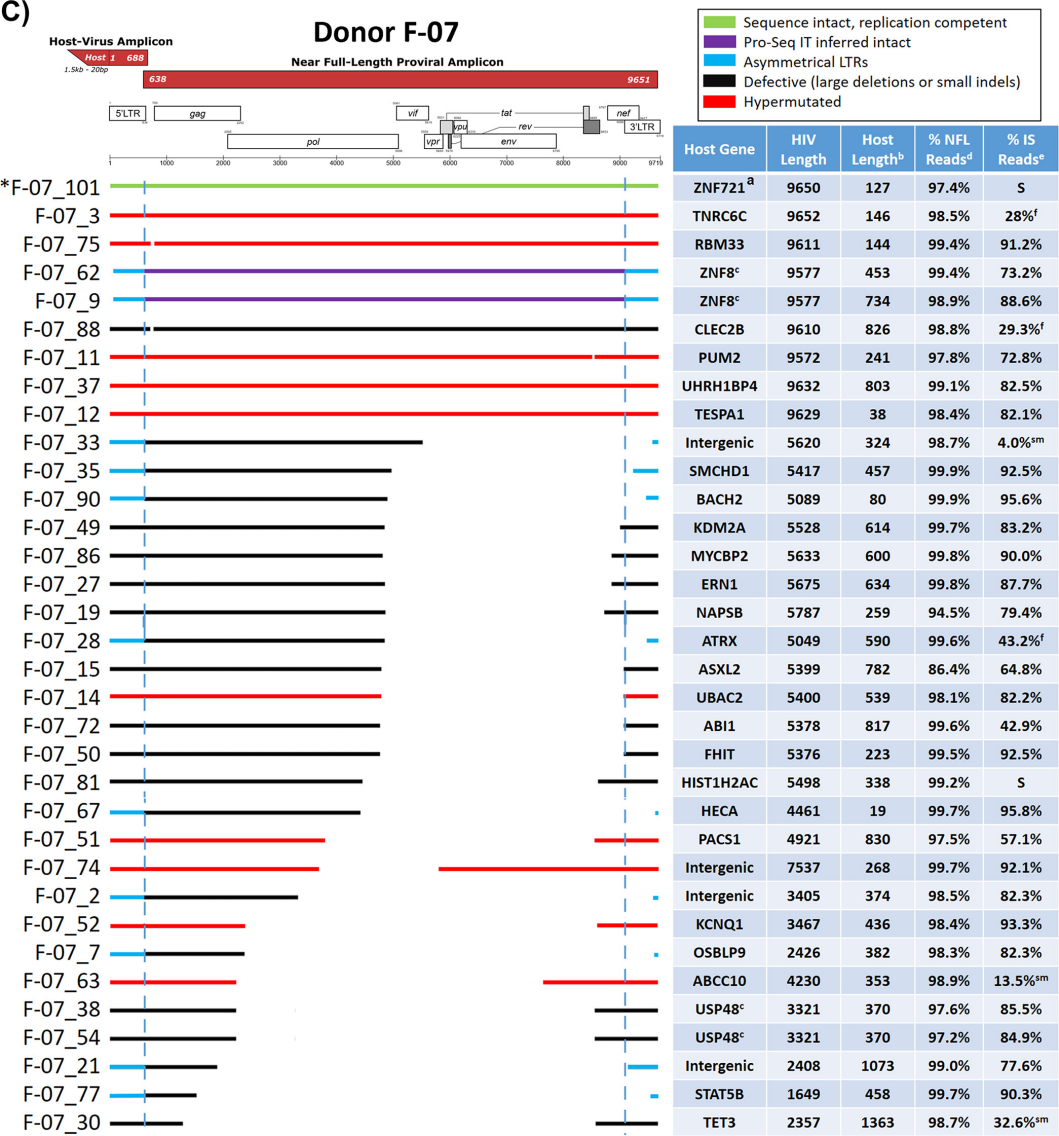

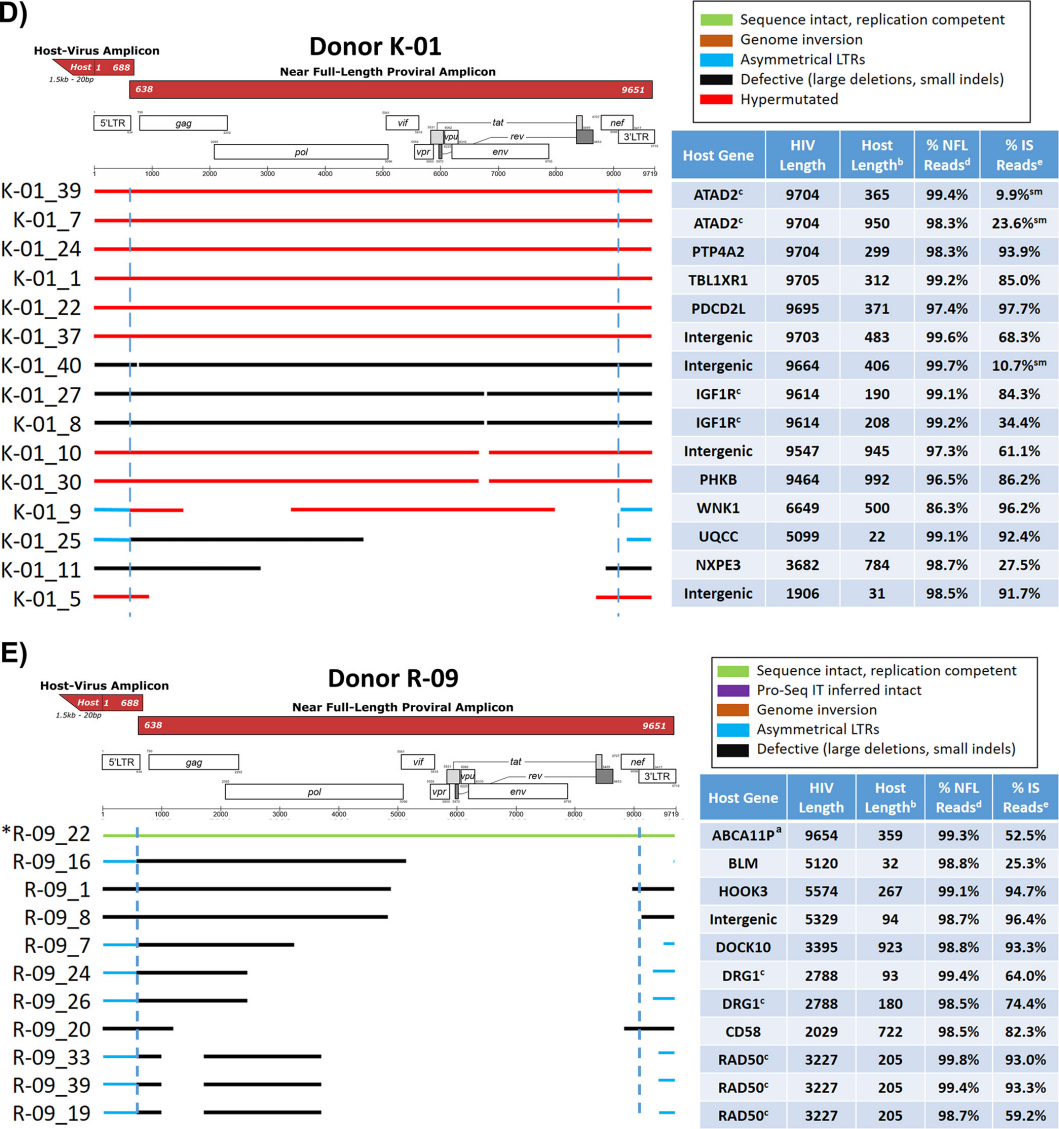
Virogram alignments of HIV-1 proviruses and integration sites from five subtype B-positive individuals. Alignments of consensus assemblies were performed using MUSCLE. Virograms for individuals C-02 (A), C-03 (B), F-07 (C), K-01 (D), and R-09 (E) are depicted. Green lines indicate proviruses determined to be replication competent by a viral outgrowth assay. Purple lines denote proviruses inferred to be sequence intact by the Proviral Sequence Annotation & Intactness Tool (ProSeq-IT) (29). Brown lines indicate regions containing proviral genome inversions. Blue lines denote proviruses with asymmetrical LTRs. Black lines indicate defective proviruses due to large deletions or small indels. Red lines indicate hypermutated proviruses determined by the Los Alamos Hypermut v2 program (*P* value of <0.05). Blue dashed lines denote LTR borders. Tables accompanying virograms correspond to the gene locations of proviral integration sites and the lengths of flanking host sequences amplified for the proviruses shown in the virograms. ^a^, replication-competent proviruses determined by a quantitative viral outgrowth assay (qVOA); ^b^, length of the amplified flanking host sequence; ^c^, proviruses in clones identified by identical proviral sequences and integration sites; ^d^, percentage of total reads utilized during the assembly of the near-full-length (NFL) consensus sequence; ^e^, percentage of total reads utilized during the assembly of the host-virus junction consensus sequence; ^f^, amplicons in which 10 ng of a faint band by agarose gel electrophoresis was sequenced; ^S^, integration sites sequenced by dideoxy sequencing only; ^sm^, amplicons in which 10 ng of DNA produced a smear by agarose gel electrophoresis but was successfully sequenced; *, proviruses for which sequence identity was validated by amplification and sequencing of the full-length provirus and flanking host DNA directly from unamplified gDNA. HIV-1 gene map art was adopted from the Los Alamos National Laboratory gene map (40).

**FIG 3:**
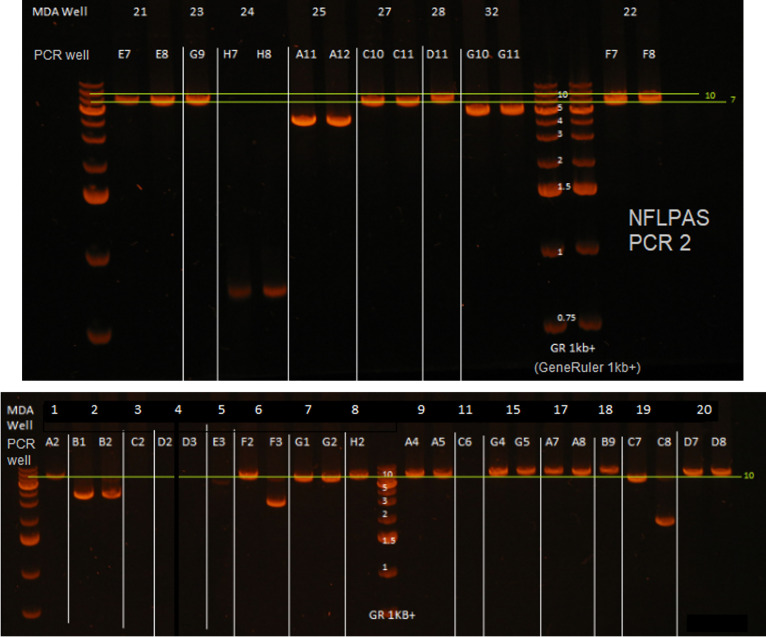
Examples of single-genome, near-full-length nested PCR amplicons. Near-full-length amplification and sequencing (NFLPAS) nested PCR was performed on MDA reactions containing single-genome HIV-1 proviruses and analyzed by 0.7% sodium borate agarose gel electrophoresis at 250 V for 30 mins using a GeneRuler 1-kb plus ladder. Shown are the results of duplicate PCR reactions from each HIV-1-positive MDA reaction. White lines separate the MDA reactions, and those MDA reactions containing only one analyzed lane are due to the duplicate reaction containing no amplified DNA by gel red dye analysis. Yellow lines denote approximate gel regions for possible intact proviruses (9 kb).

**FIG 4:**
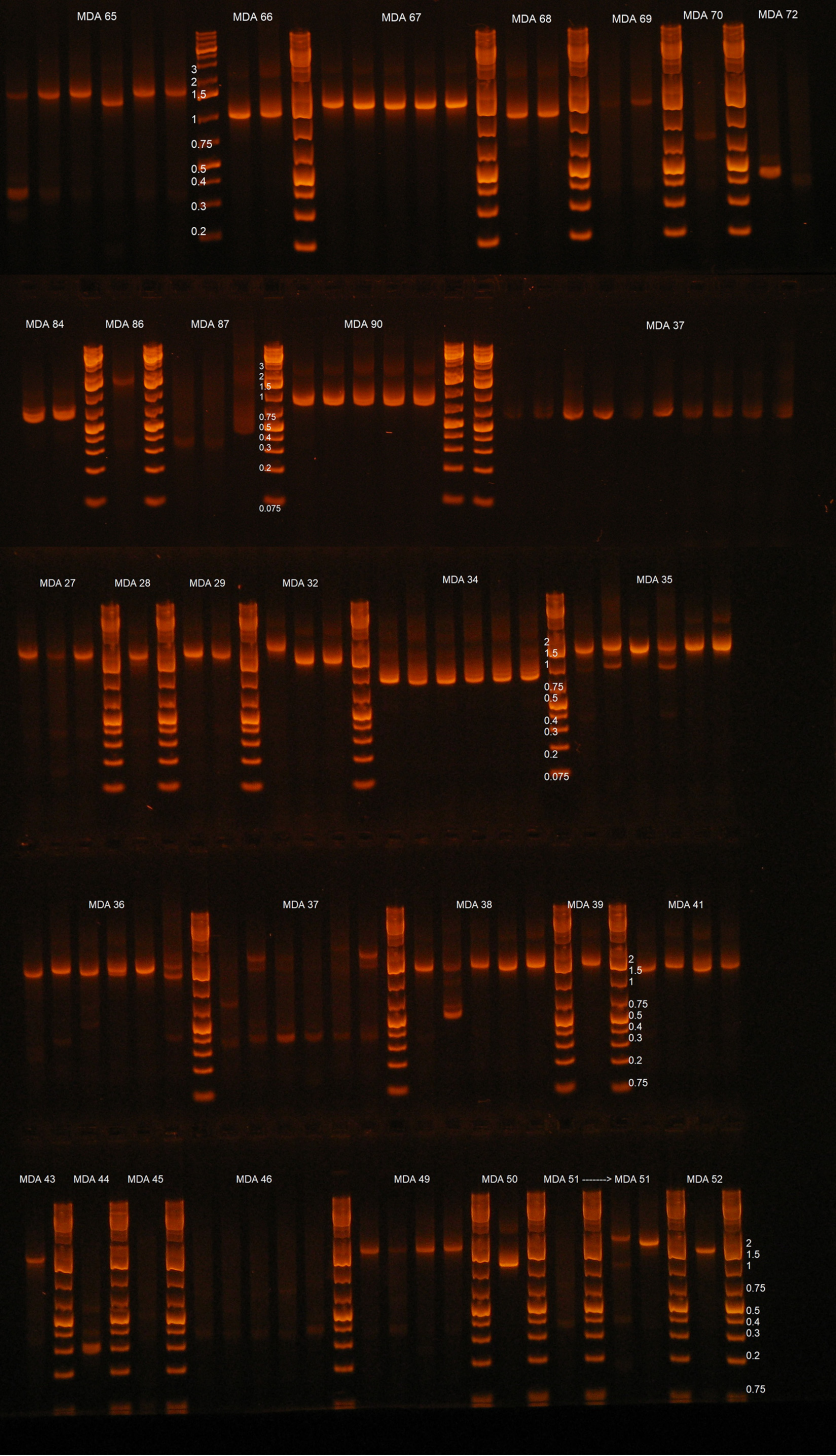
Examples of integration site (IS) nested PCR amplicons across HIV-1 host-virus junctions from various MDA reactions containing HIV-1 proviruses. IS PCR was performed on MDA reaction containing HIV-1 for which NFLPAS PCR generated a proviral amplicon. PCR wells that were positive by gel red dye screening were analyzed by 0.8% sodium borate agarose gel electrophoresis at 250 V for 15 mins with a GeneRuler 1-kb plus ladder. Each ladder separates different MDA reactions, as indicated by each lane label. In instances where PCR did not produce consistently sized amplicons or produced multiband PCR products, either incomplete restriction digestion occurred or there was more than one provirus in the MDA reaction (proviral mixture).

Table 2 shows the workflow efficiency for amplifying and sequencing ISs from MDA reactions that were positive for NFL PCR products. Matching IS amplicons are defined as amplicons containing a 5′ LTR and a *gag* leader with identical or near-identical (maximum of 3 mismatches allowed across both LTRs and *gag* leader sequences) sequence matches to the 3′ LTR and the 5′ *gag* leader of the NFL amplicon. Assay efficiencies varied by donor as a result of proviral sequence diversity, primer-binding site (PBS) deletions/polymorphisms, and variation in the number of MDA reactions containing >1 provirus before MDA (detected by proviral sequence mixtures), which were excluded from further analysis. Across all five individuals, a median of 41.2% ± 14.6% (range, 33.3% to 63.6%) of NFL-positive MDA reactions produced an integration site amplicon with 5′-LTR and *gag* sequences matching the respective overlapping NFL amplicon’s *gag* and 3’ LTR sequences. The median length of the 5′-flanking host sequence captured across these five individuals was 430 nucleotides (range, 18 to 1,363 nucleotides) (Table 2). Donor C-03 generated the smallest number of NFL-positive (*n* = 22) MDA reactions yet displayed the highest yield of concordant 5′/3′-LTR sequences (63.6%). In contrast, donor F-07 generated the most NFL-positive MDA reactions (*n* = 92), but the overall efficiency (final percent yield of proviral sequences, after accounting for proviral mixtures and proviruses with deleted IS PCR primer-binding sites) was low (36.9%). Of note, overall medians of 98.9% of NFL and 84.3% of IS sequencing reads were on target and used for the assembly of consensus sequences for all proviruses and integration sites (Fig. 2). Examples of sequenced “clean” single-band, near-full-length proviral amplicons and nullomer-mediated 5′-LTR and flanking host PCR amplicons, derived from optimized MDA reactions, are shown in Fig. 3 and Fig. 4, respectively.

**TABLE 2:** Efficiency of the individual proviral sequencing assay for five subtype B HIV-1-positive individuals^*a*^

Donor ID	No. of NFL-positive MDA reactions	No. (%) of IS-positive MDA reactions	No. (%) of proviruses without IS priming site^*b*^	No. (%) of NFLPAS-positive reactions with mixtures^*c*^	Final yield [no. (%)] of single proviral sequences^*d*^	Flanking host length (bp) (mean, range)^*e*^
C-02	34	19 (55.9)	12 (35.3)	5 (14.7)	14 (41.2)	430, 19–1,090
C-03	22	16 (72.7)	1 (4.5)	2 (9.1)	14 (63.6)	318, 18–664
F-07	92	68 (73.9)	18 (19.6)	34 (36.9)	34 (36.9)	495, 18–1,363
K-01	28	20 (71.4)	3 (10.7)	5 (17.8)	15 (53.6)	510, 22–1,137
R-09	33	15 (45.5)	5 (15.2)	4 (12.2)	11 (33.3)	328, 32–923

Median	33	19 (71.4)	5 (15.2)	5 (14.7)	14 (41.2)	430, 18–1,363

a NFL, near-full-length proviral sequences; MDA, multiple-displacement amplification; IS, integration site; NFLPAS, near-full-length proviral amplification and sequencing.

b Percentage of NFLPAS sequences containing primer-binding-site deletions for downstream integration site PCR.

c Number of MDA wells producing NFLPAS and IS amplicons with sequence mixtures.

d Number of MDA wells producing NFLPAS and IS amplicons without 5′/3′-LTR sequence identity issues.

e Mean and range of lengths in base pairs of 5′-flanking host sequences upstream of integration sites.

### Summary of individual proviruses sequenced.

**FIG 5:**
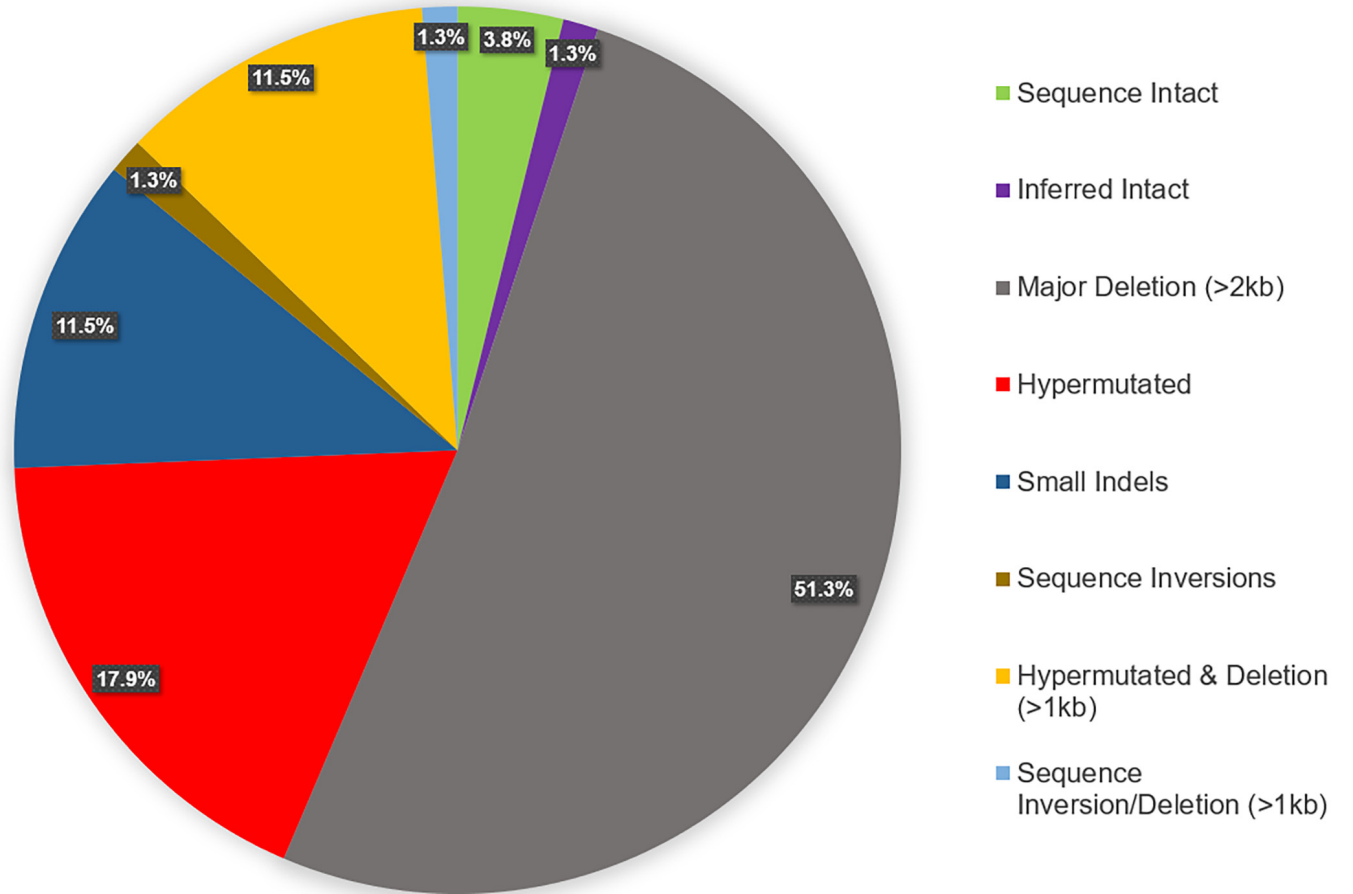
Summary of HIV-1 proviral structures sequenced from five subtype B-positive individuals. All proviruses were evaluated manually initially for the determination of sequence length and defectiveness, and sequences of full-length proviruses were evaluated for intactness by the ProSeq-IT tool (29). Of the 88 proviruses sequenced (and after removing duplicate clonal sequences from the analysis), 3.8% (*n* = 3) were sequence intact and confirmed to be replication competent by a qVOA by Halvas et al. (15), 1.3% (*n* = 1) were inferred intact by ProSeq-IT (29), 51.3% (*n* = 40) contained major genomic deletions of >2 kb in length, 17.9% (*n* = 14) were hypermutated according to the Los Alamos Hypermut v2 program (*P* value of <0.05), 11.5% (*n* = 9) both were hypermutated and contained genomic deletions of >1 kb in length, 1.3% (*n* = 1) contained sequence inversions and contained genomic deletions of >1 kb in length, 11.5% (*n* = 9) were defective due to small indels, and 1.3% (*n* = 1) contained genomic inversions.

Across the five donors, we generated a total of 209 MDA reactions containing NFL amplicons, from which 138 integration sites were amplified and sequenced, and after excluding proviral mixtures, 88 proviral genomes were assembled (Fig. 2) (28). The proportion of intact proviruses by donor varied (0 to 9.1%) (Fig. 2), with 4 intact (replication competent by viral outgrowth) or inferred intact proviruses detected in three of the five individuals (Fig. 2B, C, and E). Proviral deletions were the most commonly observed defect, found in 63.3% of sequenced proviruses and ranging in size from 90 nt to 7,798 nt. Hypermutation was the second most commonly observed defect, found in 29.1% of sequenced proviruses, ranging from 1 of 14 proviruses (donor C-03) to 10 of 15 proviruses (donor K-01). A number of the proviruses detected in four of five individuals were in clonally expanded cells (Fig. 2). Figure 5 shows a summary of the sequenced proviruses. All proviral sequences were screened for genome intactness using the ProSeq-IT tool (29). Sequence-intact proviruses across individuals confirmed to be replication competent by viral outgrowth comprised only 3.8% of proviruses detected by the IPSA. The remaining proviruses were defective due to large deletions, small indels, inversions, and hypermutations.

### Phylogenetic and clonal analyses of HIV-1 proviruses.

**FIG 6:**
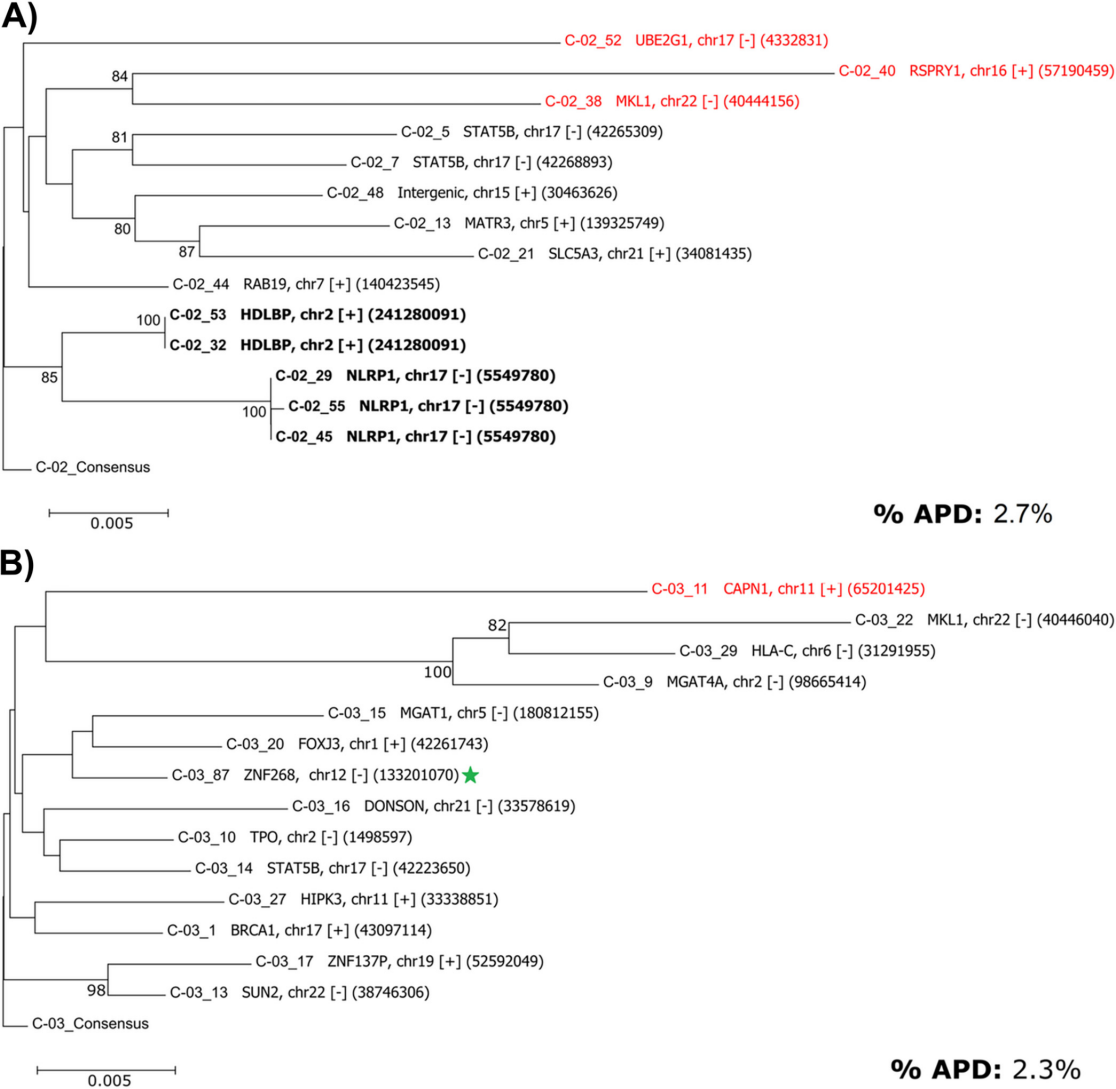

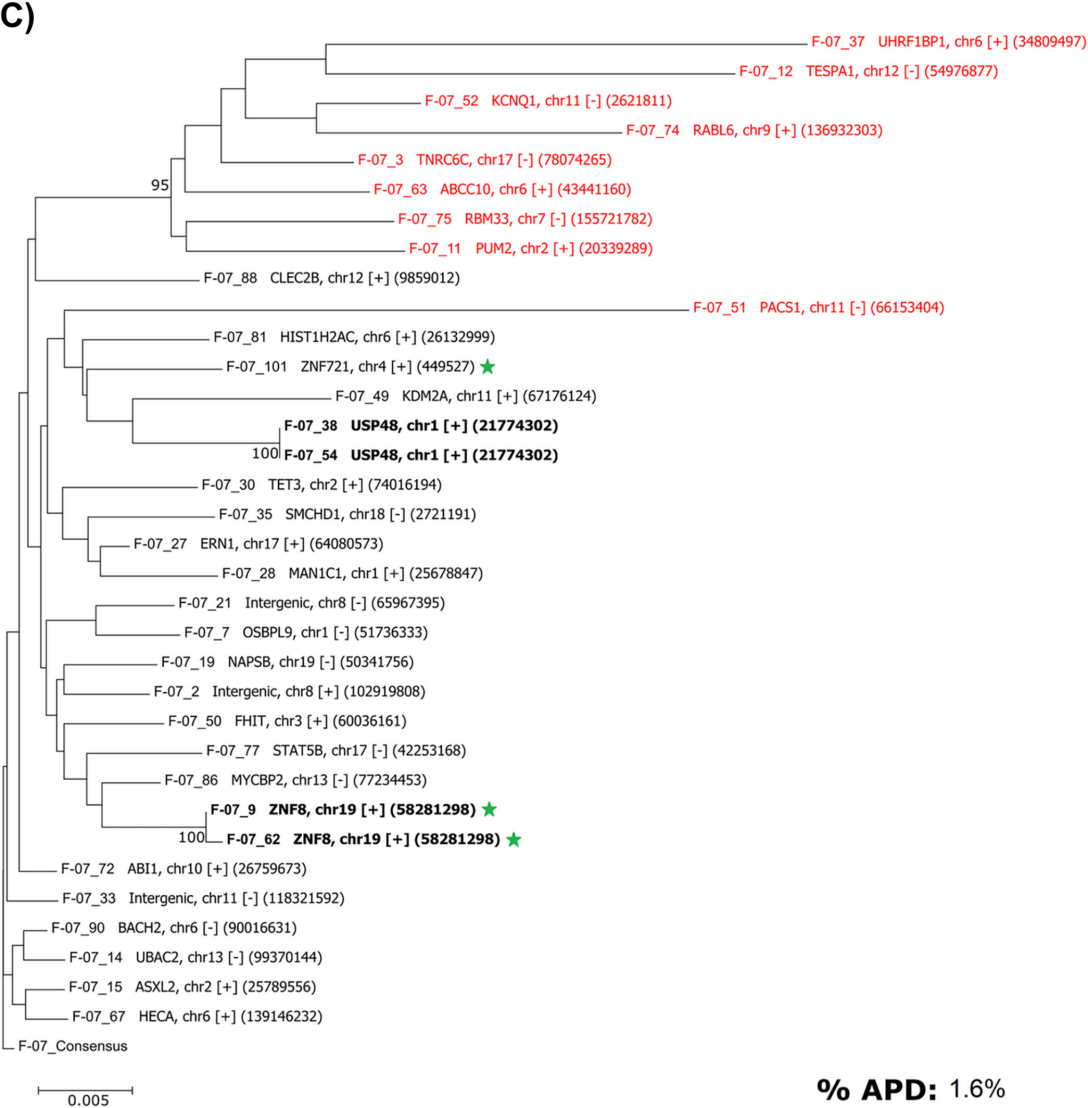

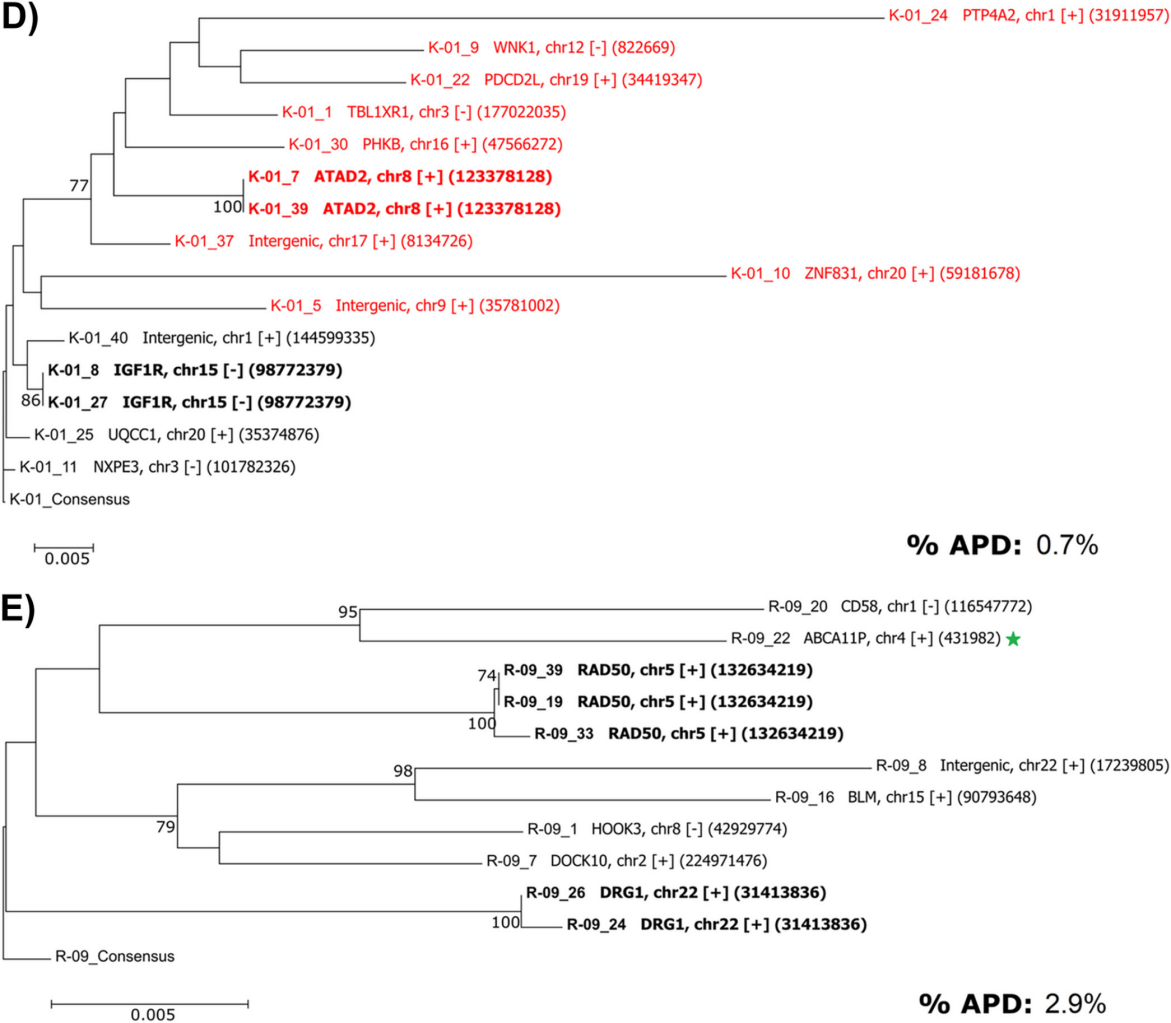
Donor-specific phylogenetic trees of genetically diverse proviruses. The first 2 kb of sequence from each provirus were aligned for comparative purposes, with gaps retained, and a neighbor-joining test of phylogeny was performed using the bootstrap method (*n* = 1,000 replicates) (MEGA v6) (39). Trees are rooted to each donor-specific consensus generated from alignments. Shown are the results of phylogenetic analyses of proviral sequences from donors C-02 (A), C-03 (B), F-07 (C), K-01 (D), and R-09 (E). Bold labels denote proviruses in clones. Red labels denote ABOBEC-hypermutated proviruses as determined by Los Alamos Hypermut v2 (*P* values of <0.05). Green stars indicate sequence-intact or inferred intact proviruses. Displayed to the right of the sequence name in each tree are the gene, chromosome (proviral orientation relative to the gene, + [with] or − [against]), and the specific location of the integration site. APD, average pairwise distance of the proviruses from each donor as determined by MEGA v6, after removing hypermutated proviruses and duplicate clonal proviral sequences.

**FIG 7:**
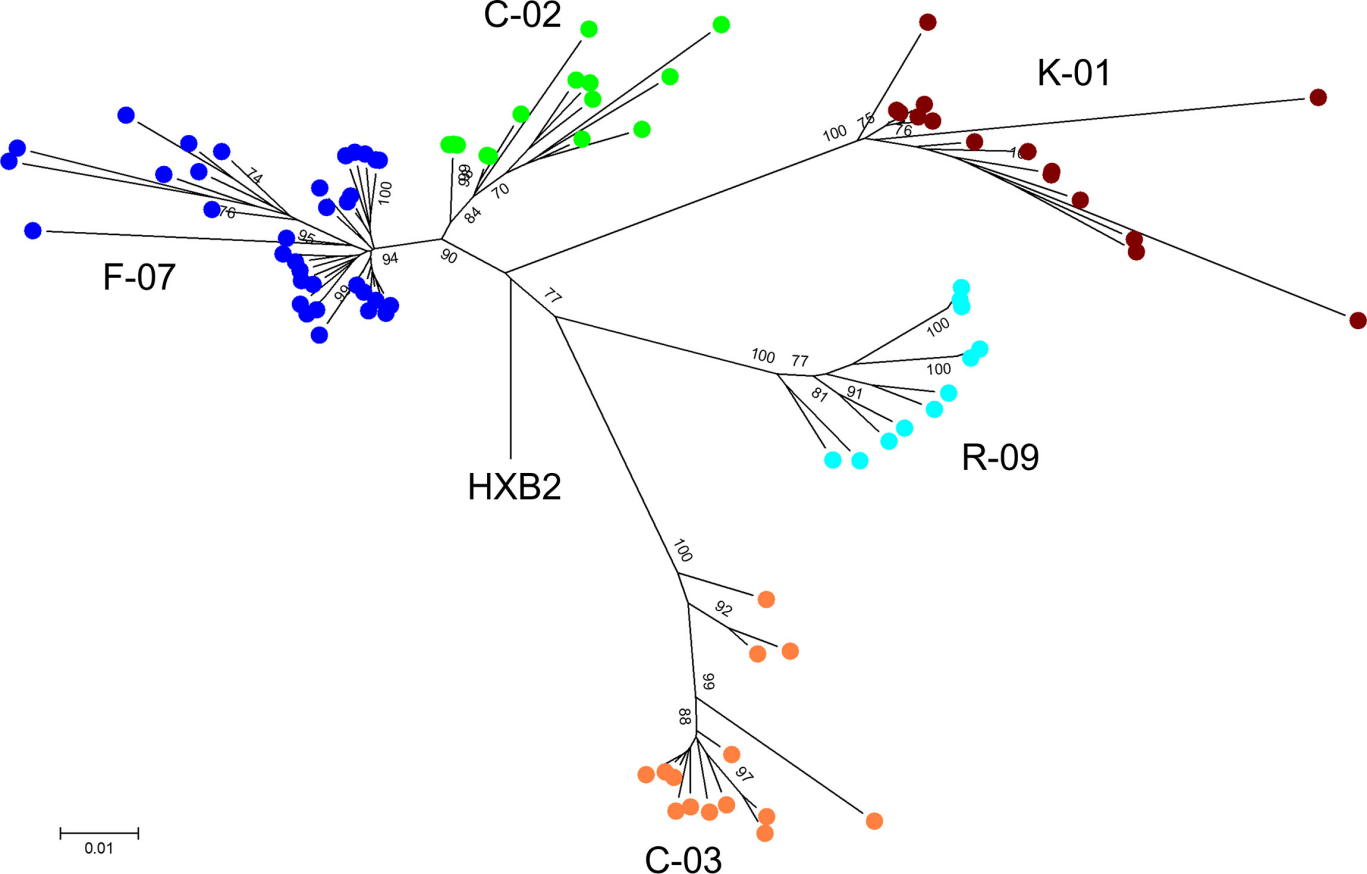
Phylogenetic tree constructed using the sequenced proviruses from all individuals showing donor-specific clustering. A neighbor-joining tree was constructed using the first 2 kb of all of the sequenced proviruses and rooted to the HXB2 consensus using MEGA v6 (39). Solid green circles represent donor C-02 sequences. Solid orange circles represent donor C-03 sequences. Solid dark blue circles represent donor F-07 sequences. Solid maroon circles represent donor K-01 sequences. Solid light blue circles represent donor R-09 sequences.

All proviral sequences from the five individuals were aligned, trimmed to the first 2 kb of the proviral genome (i.e., a common length across all sequences), and analyzed phylogenetically against subtype B HIV-1 HXB2. The IPSA generated a diverse population of proviruses, as indicated by the overall mean pairwise distances of 2.7%, 2.3%, 1.6%, 0.7%, and 2.9% for donors C-02, C-03, F-07, K-01, and R-09, respectively (Fig. 6). All sequences formed donor-specific clusters, indicating no cross-contamination between samples (Fig. 7). As described above, proviruses found in clonally expanded cells were identified in 4 of 5 donors. In donor C-02, two defective proviruses were identified in clonally expanded cells, one each integrated into the NLRP1 and HDLBP genes (Fig. 2A and Fig. 6A). No proviral sequences from donor C-02 were considered intact. Donor C-03 had no proviruses identified in clonally expanded cells; however, one provirus (C-03_87) was inferred to be intact by ProSeq-IT, which was previously determined to be clonally expanded in the ZNF268 gene by single-genome sequencing and viral outgrowth (15) (Fig. 6B). In donor F-07, two defective proviruses were identified in clonally expanded cells, integrated into the USP48 and ZNF8 genes (Fig. 6C). In donor K-01, two defective proviruses were identified in clonally expanded cells, one of which was ABOBEC3G hypermutated and integrated into the ATAD2 gene and the other of which was in the IGF1R gene (Fig. 6D). In donor R-09, two defective proviruses were identified in clonally expanded cells, integrated into the DRG1 and RAD50 genes (Fig. 6E). Interestingly, the provirus in RAD50 had a transition (purine-purine mutation) and 3 transversions (purine-pyrimidine mutation) in the 3′ LTR not present in the 5′ LTR. The provirus in RAD50 was sequenced from 3 independent MDA reactions, so these discordant nucleotides between LTRs are unlikely to be process related.

### Sequencing of both the 5′ and 3′ LTRs from individual proviruses reveals asymmetrical LTR structures.

**FIG 8:**
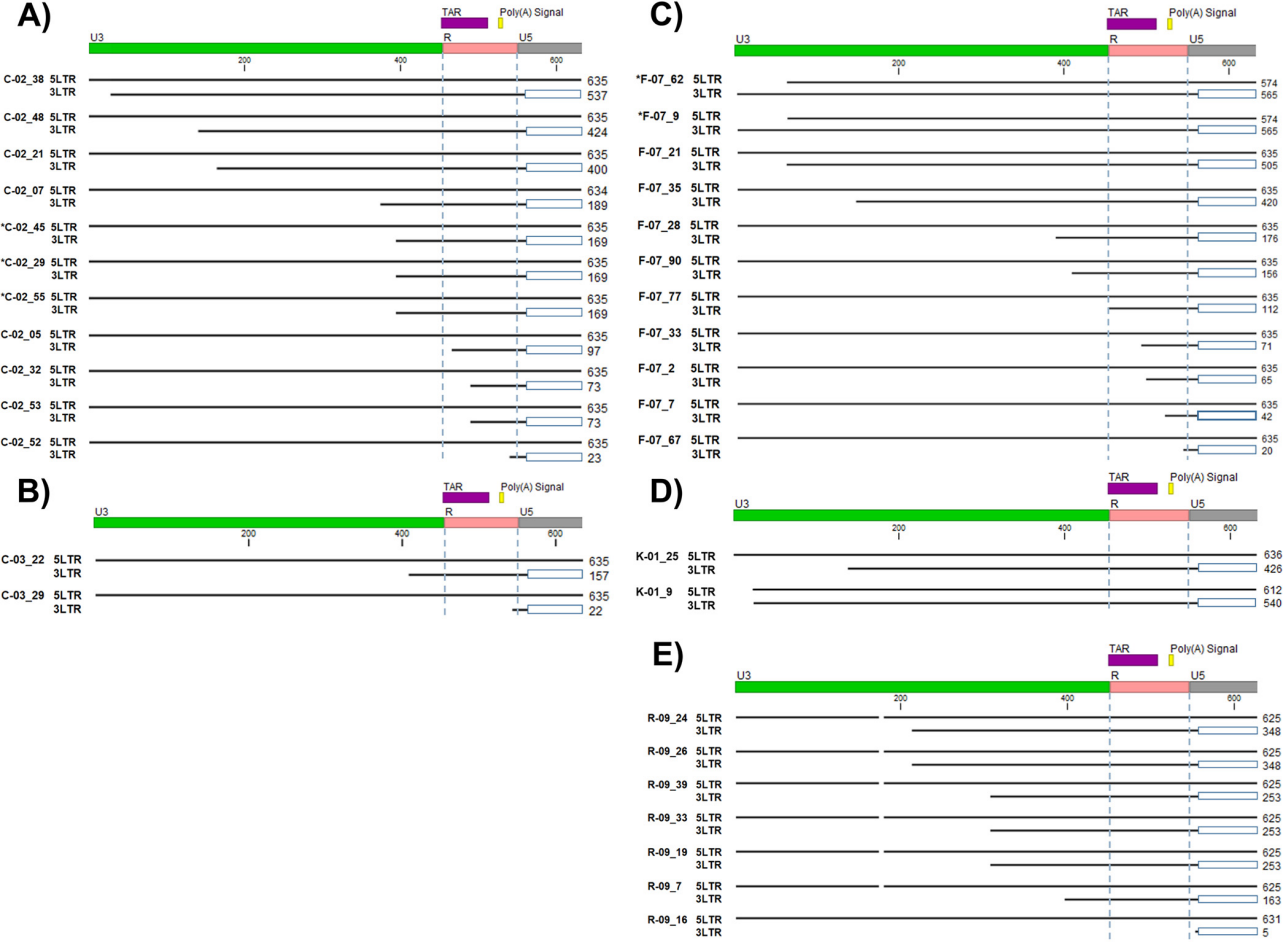
Asymmetrical 5′ and 3′ LTRs from sequenced proviruses. (A) Proviral 5′- and 3′-LTR sequences from donor C-02. (B) Proviral 5′- and 3′-LTR sequences from donor C-03. (C) Proviral 5′- and 3′-LTR sequences from donor F-07. (D) Proviral 5′- and 3′-LTR sequences from donor K-01. (E) Proviral 5′- and 3′-LTR sequences from donor R-09. The 5′- and 3′-LTR sequences were extracted from the assembled proviral consensus FASTA files and aligned using MUSCLE (28). Proviruses were defined as having asymmetrical LTRs when a large deletion was present in one LTR but not the other and the two LTRs had sequence identity within 3 mismatches. Open blue boxes indicate regions absent in the amplicon and not sequenced. Vertical blue dashed lines demarcate LTR regions R and U5. Numbers to the right of each aligned provirus indicate the length of the LTR sequence. TAR, trans-activation response element.

Among the 88 proviruses sequenced from our five individuals, 26 (29.5%) proviruses had asymmetrical LTR sequences (Fig. 8). The most common asymmetry was an intact 5′ LTR and a partially deleted 3′ LTR (*n* = 24, or 27.3% of the total proviruses or 92.3% of proviruses with asymmetrical LTRs), with the 3′-LTR deletions beginning upstream of the 3′ LTR and ending most frequently in U3. There were also 8 of 26 (30.8%) proviruses with asymmetrical LTRs having complete 3′-LTR U3 and partial R deletions, and 1 of 26 proviruses (3.8% of proviruses with asymmetrical LTRs) had the entire 3′-LTR U3 and R regions deleted (Fig. 8E, R-09_16). Rare proviruses with sequence-intact 3′ LTRs and partially deleted 5′ LTRs were also identified (*n* = 2; 7.7% of asymmetrical proviruses), although deletions found in the 5′ LTR were much shorter (<100 nt) than those found in the 3′ LTR (Fig. 8C, F-07_62, and Fig. 8D, K-01_9). One provirus (K-01_9) was identified that had a 24-bp deletion at the beginning of the 5′-LTR U3 but an additional deleted nucleotide in the 3′-LTR U3 (Fig. 8D, K-01_9). Additionally, 4 proviruses (4.5% of the total proviruses) contained 5′ LTRs that were sequence intact and 3′ LTRs with deletions encompassing the transcription termination/polyadenylation signal (Fig. 8, samples C-02_52, C-03_29, F-07_67, and R-09_16). These data caution against previous assumptions of proviral LTR symmetry and may contribute to the underrepresentation of proviral burden estimated by LTR-targeted amplification methods.

### Analysis of proviral integration sites.

**FIG 9:**
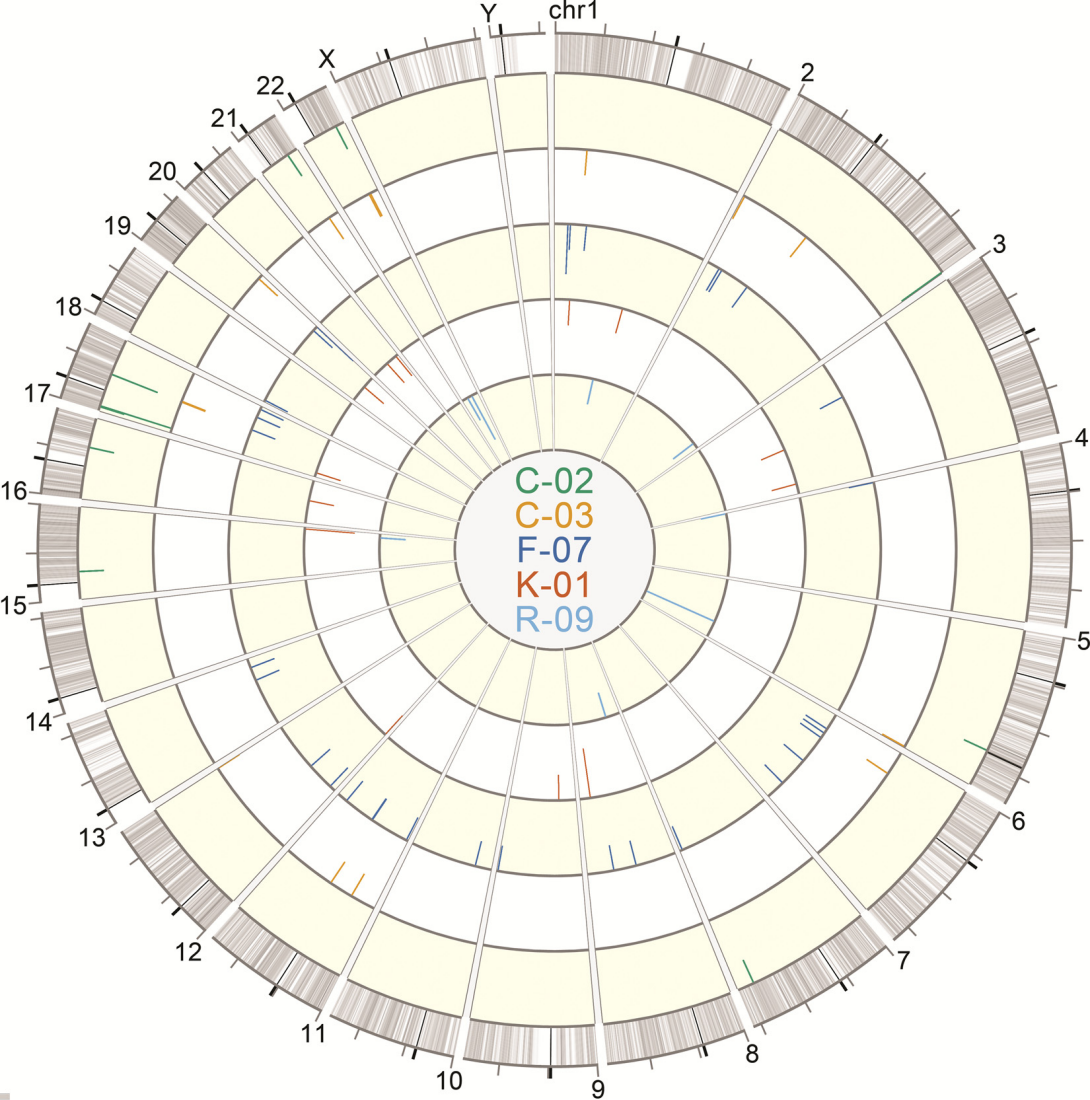
Circos plot of HIV-1 integration sites of sequenced proviruses. Sequencing reads containing the 5′-host-virus junction were used to extract integration sites from sequencing FASTQ files for alignment against the hg38 consensus genome using the UCSC BLAT tool (37). The Circos plot was generated using Vgas software (38). Each ring depicts integration sites of sequenced proviruses from each donor: C-02 (green), C-03 (orange), F-07 (dark blue), K-01 (red), and R-09 (light blue). The lengths of the tick marks are proportional to the number of times that an identical integration site of a specific provirus was independently amplified and sequenced.

**FIG 10:**
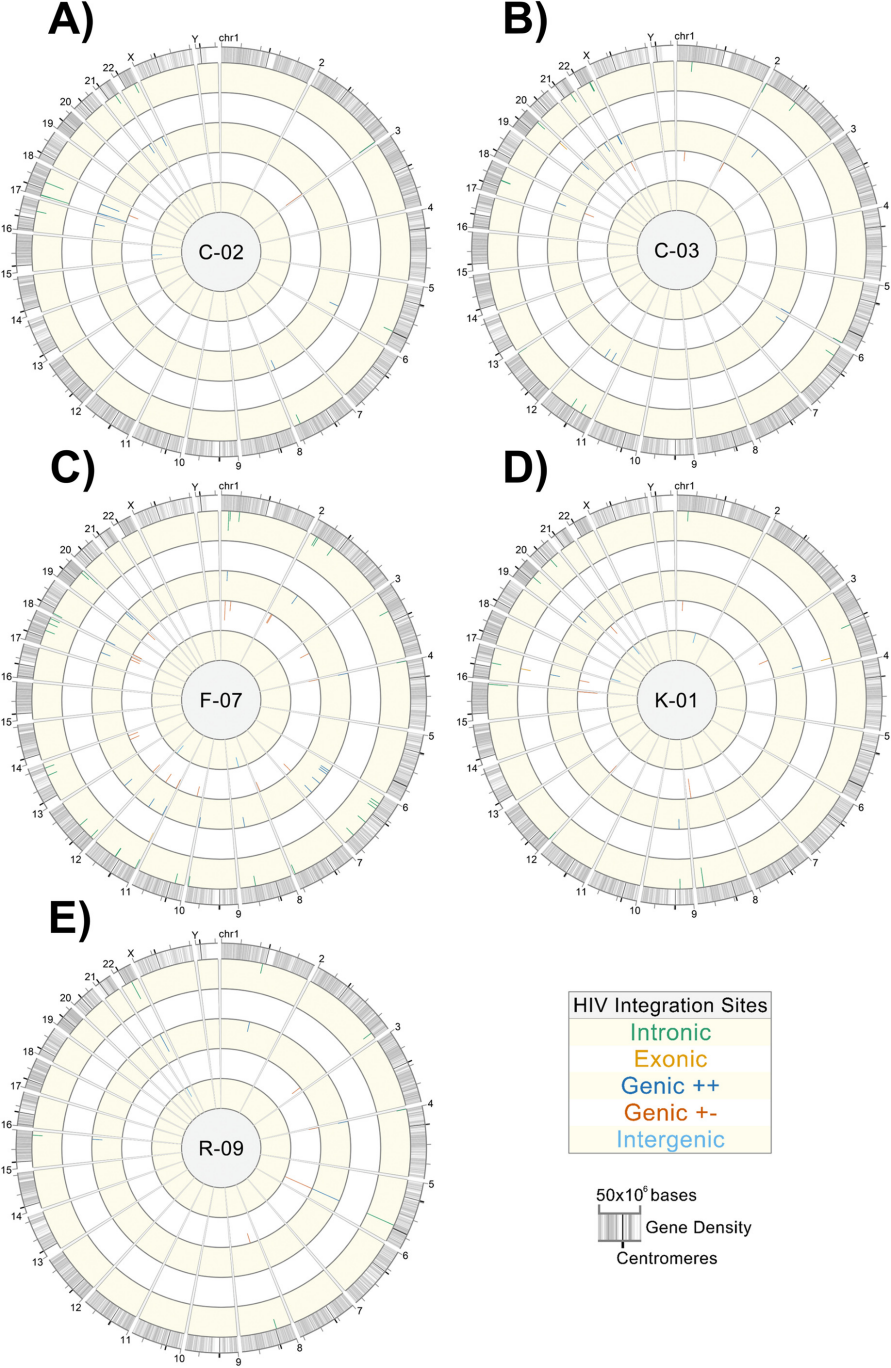
Donor-specific integration site Circos plots. Sequencing reads containing the 5′-host-virus junction were used to extract integration sites from sequencing FASTQ files for alignment against the hg38 consensus genome using the UCSC BLAT tool (37). Circos plots were generated using Vgas software (38). (A) Donor C-02; (B) donor C-03; (C) donor F-07; (D) donor K-01; (E) donor R-09. Each plot depicts all amplified and sequenced proviruses and linked integration sites within a specific donor, represented by tick marks along the rings. Rings from outermost to innermost show proviruses integrated into introns, exons, genic locations in the same orientation as that of the gene, genic locations in the opposite orientation as that of the gene, and intergenic regions, respectively. The lengths of the tick marks are proportional to the number of times that an identical integration site for a given provirus was independently sequenced.

Circos plots were generated to show the chromosomal locations of integration sites found in the donors, with each donor being depicted as a separate ring (Fig. 9). Figure 10 shows the proviral landscape separately for each donor. Across all 5 individuals, after removing duplicate clonal sequences for analysis, 76.9% (*n* = 60) of the proviruses were integrated into introns, 5.1% (*n* = 4) were in exons, and the remaining 9.0% (*n* = 7) were integrated into intergenic regions (See Table S1 in supplemental materials). For proviruses located near genes, 44.9% (*n* = 35) were integrated in the same orientation as that of the gene (Genic + +), whereas 46.2% (*n* = 36) were in the opposite strand (Genic + −).

### Validation of proviral sequences using gene- and HIV-specific nested PCR.

A subset of proviral genome and integration site sequences acquired through the IPSA workflow was validated by integration-site-specific proviral PCR, as previously described, in which gene-specific primers paired with provirus-specific primers were used to amplify and sequence the full-length provirus in two fragments from unmodified gDNA extracted from PBMC (15). The identification of the same provirus integrated into the same location in two different samples requires the provirus to be in a cell clone. Accordingly, a subset of 4 proviruses, each determined to be in clonally expanded cells, was amplified and sequenced using gene- and HIV-specific PCR primers. The replication-competent provirus (F-07_101) from donor F-07, integrated at hg38 chromosome 4 reference position 449527 (chr4:449527) (ZNF721 gene), was amplified and sequenced from unmodified gDNA. A comparison of the gene- and HIV-specific PCR-generated sequence with the MDA-derived sequence showed a perfect match (0 differences out of 9,777 bp sequenced) and an identical integration site. The defective proviruses with asymmetrical LTRs from donor C-02 (C-02_29, C-02_45, and C-02_55) integrated at hg38 reference position chr17:5549780 (NLRP1 gene) were also amplified and sequenced independently three times from PBMC gDNA. The integration-site- and HIV-specific sequence showed perfect sequence identity with two of the MDA-derived sequences (the third sequence had an A→T transversion at nucleotide position 1197). The replication-competent provirus (C-03_87) integrated into the ZNF268 gene at hg38 reference position chr12:133201070 from donor C-03 was amplified and sequenced, yielding sequences with near-perfect matches to the MDA-derived sequence and integration site (2 mismatches out of 9,697 bp). Finally, the replication-competent provirus (R-09_22) integrated at hg38 reference position chr4:431982 in the ABCA11P gene from donor R-09 was amplified and sequenced from four independent gene-specific PCR reactions and had perfect sequence matches (0 mismatches out of 9,743 bp sequenced) with the MDA-derived proviral and integration site sequences.

As noted above, we found many proviruses with asymmetric LTRs. To validate the accuracy of these findings, we performed nullomer-mediated PCR across the 3′-LTR deletions and host-virus junctions for a subset of proviruses containing asymmetrical LTRs. For all 8 proviruses with asymmetrical LTRs evaluated, nullomer-mediated PCR produced proviral sequences that were identical to those produced by the original IPSA (C-02_38, C-02_52, C-03_22, C-03_29, R-09_7, R-09_19, R-09_33, and R-09_39).

## DISCUSSION

Here, we report a new method to amplify and sequence individual HIV-1 proviruses (IPSA) that was applied to PBMC samples from five HIV-1-positive individuals with subtype B infection on ART (Table 1) and revealed intact and defective proviruses, previously unrecognized asymmetrical proviral structures, and identical proviruses and integration sites in clonally expanded cells. The samples analyzed from 3 of the 5 donors (C-02, C-03, and R-09) were previously reported to have non-suppressible, clinically detectable viremia (plasma HIV-1 RNA level of >20 copies/mL) originating from large cell clones (15) carrying replication-competent proviruses, which occurs infrequently among persons on long-term ART. Despite the presence of these large, virus-producing clones, which constituted a very small fraction of all the proviruses in these donors, the characteristics of the proviruses identified by the IPSA, with most being defective from a variety of mechanisms, are in keeping with what has been observed previously by others in the field analyzing samples from persons on long-term ART (8, 21, 22, 29).

A subset of the different types of proviruses in cell clones was confirmed to be present in unmodified gDNA in PBMC by host gene- and HIV-specific PCR, validating the accuracy of the IPSA method. Overall, the yield of near-full-length proviruses and their corresponding integration sites was 41% of MDA reactions that contained an amplifiable near-full-length provirus. Medians of 99% and 84% of next-generation sequencing reads were appropriate to include in the generation of consensus sequences from near-full-length proviral and integration site amplicons, respectively, indicating the high specificity of the method (few off-target reads). We believe that this on-target amplification and sequencing efficiency is the highest reported to date for individual proviruses and their paired integration sites, and the throughput and validated accuracy of this assay will be sufficient to support evaluations of ongoing and future curative interventions.

Key steps in the workflow enable the efficiency and accuracy of the IPSA. First, the optimization of the MDA step used to amplify single proviral templates prior to PCR improves the product yield (Fig. 1 to Fig. 4 and Table 2). The addition of trehalose to the MDA reaction and a higher reaction temperature (40°C versus the standard 30°C) likely reduce random-primer-derived template-independent DNA amplification during MDA (24). Furthermore, by performing MDA with a 2:1 ratio of hg19 random decamers to random nonamers, we propose that more full-length copies of the provirus are generated during MDA due to greater priming occurring within human gDNA and less random priming occurring within the provirus, resulting in improved consistency and efficiency of downstream long-range PCR.

Second, nullomer-mediated PCR yielded high specificity and efficiency for amplifying the 5′-LTR and flanking integration site from individual proviruses, without the need for gene-specific PCR primers. Specifically, nullomer-mediated integration site amplification routinely produced over 150 ng of single-band amplicons, which were sequenced by Sanger and/or NGS methods without the need for gel purification. Most (84% [interquartile range {IQR}, 60.2 to 92.5%]) NGS reads from integration site amplicons were on target and used for consensus generation, which allows highly multiplexed sequencing runs and reduced sequencing costs. Nullomer-mediated PCR can also amplify longer reads of both the provirus and flanking host DNA as one amplicon than with previous methods (Fig. 2) (10, 23, 25). Longer reads of the host sequences flanking the integration site improve the ability to pinpoint the specific integration sites in more ambiguous chromosomal regions.

A major challenge for PCR-based technologies is to accurately characterize 10,000 bp of the HIV-1 target sequence in a background of 3 × 10^9^ off-target base pairs per cell and to avoid the generation of proviral artifacts through mispriming and recombination during PCR. Accordingly, the sequence validity of IPSA-identified proviruses was assessed using integrant-specific PCR (also known as “host-to-full-length-provirus-to-host” [HFH] amplification and sequencing) to amplify the proviruses from PBMC gDNA. HFH amplification of the three clonally expanded, replication-competent proviruses from PBMC DNA from donors C-03, F-07, and R-09 produced sequences identical to the IPSA-derived sequences with the exception of C-03_87, which had 2 mismatches out of 9,697 bp. Additionally, HFH PCR of the C-02 NLRP1 integrant with asymmetrical LTRs produced sequences identical to those generated by the IPSA. Additional validation of the IPSA workflow has also been performed with the ACH2 cell line by amplifying and sequencing the provirus integrated into the NT5C3A gene (hg38 reference position chr7:33019786), with matches identical to what has been reported by other laboratories (30) (data not shown [available in GenBank and the Sequence Read Archive {SRA} submissions]).

For proviruses with asymmetrical LTRs, short-range nullomer-mediated PCR using provirus-specific primers across the 3′-host-virus junction was performed using the existing nullomer-ligated IS libraries to exclude artifact formation during long-range NFL PCR and produced sequences identical to those generated by the original IPSA. Taken together, these data indicate that the IPSA does not generate artifacts when characterizing both replication-competent proviruses and defective proviruses with or without asymmetrical LTRs. Similar validations have not been reported for other methods characterizing proviruses and their integration sites.

**FIG 11:**
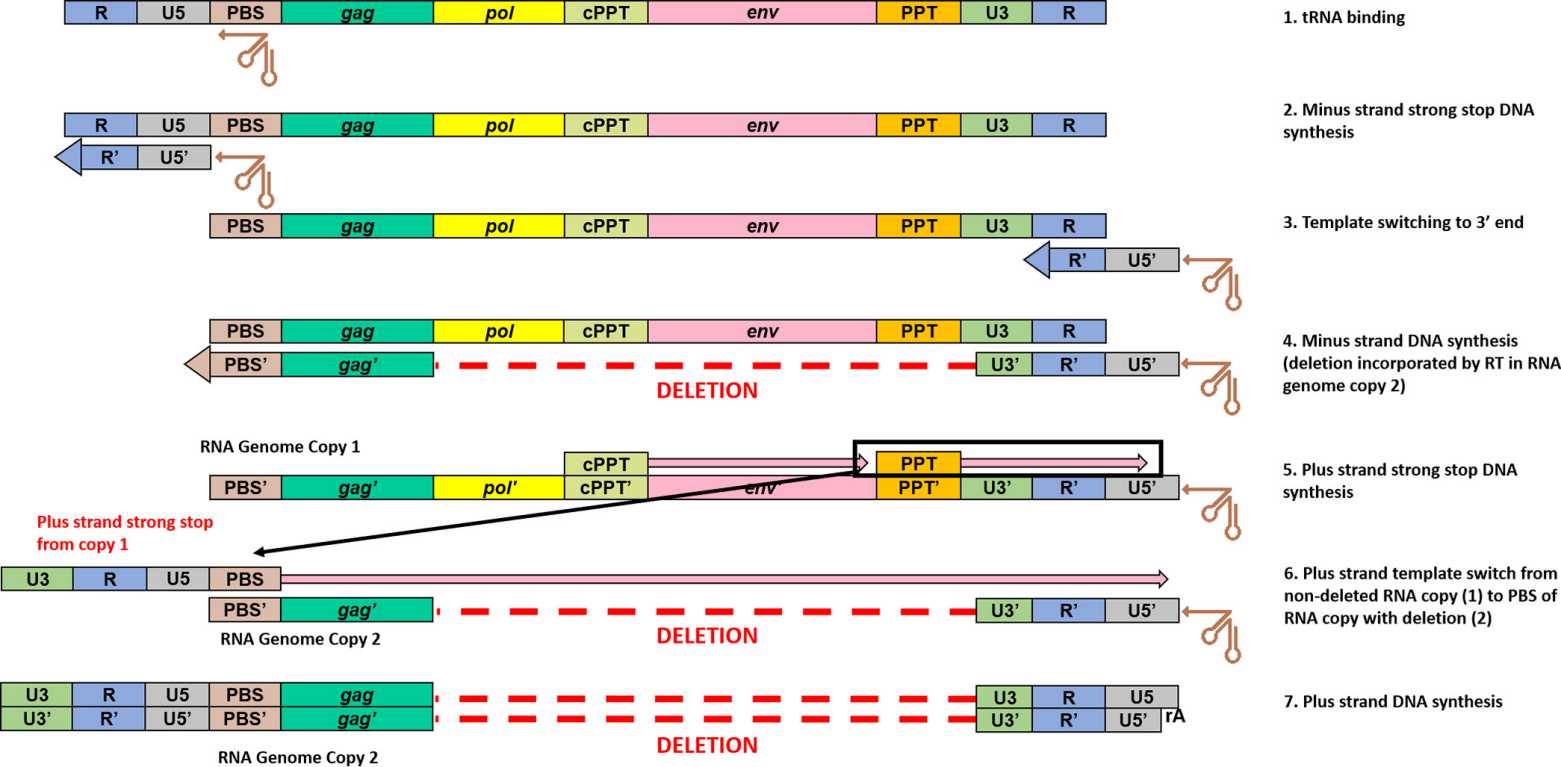
Proposed mechanism for the generation of proviruses with asymmetrical LTRs (an intact 5′ LTR with a deletion in the 3′ LTR). (1) At the initiation of reverse transcription, the tRNA is annealed to the primer-binding site (PBS). (2) RT initiates minus-strand DNA synthesis from the tRNA 3′-OH and copies the U5 and R portions at the 5′ end of the genome. (3 and 4) Template switching of the minus-strand strong-stop DNA occurs by annealing of the newly synthesized 5′-R to the 3′-R sequence (3), which then allows minus-strand DNA synthesis to proceed (4). (5) HIV-1 polypurine tract (PPT) tracts are resistant to RT RNase H degradation and thus allow priming and initiation of plus-strand DNA synthesis, using the new minus-strand DNA copy as a template. (6) After RT copies the 3′-U3, -R, -U5, and -PBS regions, template switching can occur where the newly copied 3′-PBS sequence of the intact copy of plus-strand strong-stop DNA (shown here from RNA genome copy 1) anneals to the 5′-PBS sequence of the RNA genome copy containing an internal deletion. (7) Plus-strand DNA synthesis proceeds and copies the minus strand containing the internal genomic deletion.

To our knowledge, this is the first report of sequences from both the 5′ and 3′ LTRs of individual proviruses derived from clinical samples, and our findings show that both LTRs of a provirus are not always identical in sequence and/or structure (Fig. 2 and Fig. 8). Although the majority of proviruses had LTRs with perfect or near-perfect sequence matches, 29.5% had structural differences between the two LTRs, with the 3′ LTR commonly having large deletions extending upstream into the provirus that were not present in the 5′ LTR. Additionally, 4.5% (*n* = 4) of the proviruses reported here had intact 5′ LTRs and deletions of the poly(A) signals in the 3′ LTR. We propose that the creation of asymmetrical LTRs occurs during reverse transcription by a misalignment mechanism (31) (Fig. 11). During minus-strand DNA synthesis, RT can dissociate from the template strand and reassociate downstream, deleting the intervening region. Since HIV-1 virions package two RNA genome copies, the plus-strand strong-stop DNA produced by copying one RNA genome copy can template switch onto the 5′ PBS of the other RNA genome copy containing the deletion starting in the 3′ LTR, allowing the completion of plus-strand DNA synthesis and the subsequent integration of the asymmetric provirus (Fig. 11).

HIV-1 proviruses with asymmetrical LTRs have several important implications. First, analysis of proviruses using only one LTR sequence is likely to be problematic, especially when assessing clonality, as the sequence variations between LTRs may complicate the accurate classification of clones. The frequent occurrence of asymmetrical LTRs is also likely to confound the quantification of integrated HIV-1 DNA by LTR-specific quantitative PCR (qPCR) due to the heterogeneity in sequences between LTRs. Furthermore, amplification of proviruses with asymmetrical LTRs may pose challenges for near-full-length amplification using primer-binding sites in the LTR because sequences present in the 5′ LTR may be absent in the 3′ LTR and vice versa. Finally, the integration of defective proviruses with intact 5′ LTRs and deleted 3′-LTR poly(A) signals may facilitate readthrough transcription and the activation of oncogenes, as has been observed for other retroviruses (32, 33).

Although the IPSA has improved on previous methods used for sequencing HIV-1 proviruses, there are limitations to its current iteration. The need to perform MDA at a proviral endpoint dilution is inherently inefficient; by design, ≥70% of MDA reactions are expected to be negative for proviruses. Additionally, if samples are not completely homogeneous (due to clumping of high-molecular-weight gDNA) or are insufficiently diluted prior to MDA, a significant proportion of MDA reactions will contain proviral mixtures, which will reduce the overall efficiency of characterizing single-genome proviruses, as was seen in this data set, with a median of 14.7% of HIV-containing MDA reactions containing a proviral mixture. Donor F-07 in this study had the most amplified proviruses (*n* = 97) but also had the lowest overall efficiency (36.9%) because dilution to a single proviral endpoint was incomplete (Table 2). Alternative methods such as overlapping, shorter-range nested PCR or qPCR targeting highly conserved sequences can be used to refine the proviral endpoint, although the additional steps can reduce the assay throughput. In addition, PCR assays such as the intact proviral DNA assay (IPDA) that target multiple parts of the genome simultaneously can be used to screen MDA reactions for proviruses that are more likely to be sequence intact (34).

Another limitation is that the overlap in proviral sequences between the NFL and IS amplicons is limited to 49 bp of the *gag* leader, which could reduce confidence in matching a provirus to an integration site if a mixture of proviruses was present within the same MDA reactions. However, the use of the smallest overlap possible was intentional due to the high degree of conservation surrounding the integration site PCR primer-binding sites and to avoid potential internal proviral deletions. A comparison of the 5′- and 3′-LTR sequences by ProSeq-IT showed that nearly all of our sequenced nonhypermutated proviruses, with at least 20 nucleotides of the 3′ LTR present (*n* = 68), had perfect or near-perfect sequence identity between the LTRs (perfect match, *n* = 50; 1 mismatch, *n* = 12; 2 mismatches, *n* = 2; 3 mismatches, *n* = 4), suggesting that the two amplicons originated from the same provirus and not from a proviral mixture. One to three mismatched bases were likely attributed to PCR error or LTR mutations introduced during the synthesis of the LTRs in the viral replication cycle. Nevertheless, increasing the overlap of proviral sequences between the two amplicons would further increase confidence that proviral mixtures were excluded. Additionally, like all PCR-dependent workflows, the IPSA workflow will not amplify proviruses that contain PCR primer-binding-site deletions; thus, proviruses missing U5 in either LTR will not be detected, and those missing *gag* leader sequences will not be amplified by integration site PCR. A final limitation is that IPSA does not provide a sequence for the last 69 bp of the 3′-LTR U5 region. However, by using nullomer-mediated PCR to amplify across the 3′ LTR and the flanking host-virus junction during validations of the proviruses with asymmetrical LTRs, the remaining 69 bp in these samples and their 3′ integration site were sequenced, suggesting that with some minor modifications, true full-length proviral and both 5′- and 3′-integration site sequencing can be achieved with the IPSA. Of note, although several steps of the IPSA workflow are currently automated, higher throughputs are possible with additional liquid-handling automation and a laboratory information management system (LIMS).

In summary, the IPSA improves on previously reported methods for characterizing single proviruses. Amplification of the host-virus junction and the remaining provirus (minus 69 bases in the 3′-U5 region) in two overlapping amplicons from the same MDA reaction simplifies the workflow. The high specificity and efficient amplification of nullomer-mediated PCR allow highly multiplexed integration site sequencing, thereby improving sequencing throughput and reducing sequencing costs. Sequencing of both LTRs of individual proviruses has provided new insights into HIV-1 proviral structures, including frequent (29.5%) asymmetry of 5′ and 3′ LTRs. Further modifications of the assay are in progress to include the 3′-host-virus junction to complete sequencing of entire proviruses and flanking host regions. Such a level of detail is needed to fully characterize proviruses that persist despite long-term antiretroviral therapy and to evaluate the impact of current and future curative interventions on the proviral reservoir.

## MATERIALS AND METHODS

### Study participants and sample collection.

Peripheral blood mononuclear cell (PBMC) samples were obtained from large-volume blood draws (180 mL) or leukapheresis, from April 2014 to April 2017. Samples were obtained under a human research protocol (PRO10070203) approved by the University of Pittsburgh’s institutional review board. All participants provided written informed consent before enrolling in the study.

### Workflow of the individual proviral sequencing assay (IPSA).

The workflow for amplifying single HIV-1 proviruses is outlined in Fig. 1. In brief, gDNA was extracted from 1.25 × 10^6^ PBMCs serially diluted to <1 provirus per NFL PCR reaction (≤30% positive for near-full-length [NFL] amplicons). Multiple-displacement amplification (MDA) was performed, and the near-full-length provirus and 5′-LTR-host-virus junction were PCR amplified and sequenced using both Sanger and Illumina platforms. Detailed descriptions of each step in the workflow are provided below.

### Extraction of genomic DNA from HIV-1-infected cells.

gDNA was extracted as previously reported, with the following modifications (15, 35). Briefly, 1.25 × 10^6^ PBMC were pelleted at 500 × *g* for 15 min at 4°C in a 1.5-mL microcentrifuge tube, the supernatant was removed, 600 μL of lysis buffer was added, the tube was vortexed briefly for 3 to 4 s, and cells were incubated in lysis buffer for 10 min at room temperature (15). After incubation, 600 μL of 100% isopropanol was added to the tube(s), and the tube(s) was inverted >30 times for sufficient mixing (to minimize gDNA shearing) and centrifuged at 21,100 × *g* for 20 min at 10°C, after which the supernatant was removed. The pellet was washed by the addition of 1 mL of ice-cold 70% ethanol and mixed by inverting the tube >30 times, the contents were centrifuged at 21,100 × *g* for 20 min at 10°C, the supernatant was carefully removed, and the ethanol wash was repeated twice more. The gDNA pellet was air dried for 15 min at room temperature and resuspended in 200 μL of ice-cold 5 mM Tris-HCl (pH 8.0) by careful pipette mixing of the DNA.

### Single proviral endpoint determination.

Extracted gDNA from PBMCs was serially diluted 3-fold, and 2 μL from each dilution of gDNA was spread across rows of a 96-well plate. NFL proviruses were amplified by nested PCR on the entire plate under the conditions described below. NFL PCR amplicons can range in size from <1 kb up to 9 kb, and the dilution producing ≤30% positive PCR replicates (regardless of amplicon size) was selected for downstream MDA.

### Multiple-displacement amplification.

gDNA was diluted to a single proviral endpoint (as described above) in a final volume of up to 210 μL in molecular-grade water. Two microliters was seeded across all 96 wells of a 96-well plate, and MDA was performed as previously described, with the following modifications (24, 26). Briefly, gDNA was denatured with 2 μL of fresh 0.4 M KOH–10 mM EDTA for 3 min at room temperature and then neutralized with 2 μL of 0.3 M Tris-HCl (pH 7.5)–0.2 M HCl–0.75 M trehalose on an ice block for 1 min. After neutralization, 19 μL of ice-cold MDA master mix [final reaction mixture concentrations, including denaturation and neutralization components, of 10 mM Tris-HCl (pH 7.5), 10 mM MgCl_2_, 10 mM (NH_4_)_2_SO_4_, 6 mM dithiothreitol (DTT), 100 ng/μL bovine serum albumin (BSA), 3 mM deoxynucleoside triphosphates (dNTPs), 0.6 M trehalose, 20 μM phosphorothioate-terminated random nonamers, 40 μM hg19-specific random decamers (See Table S2 in supplemental materials), and 0.3 U/μL phi29 polymerase] was added to the plate on the ice block, and the sample was mixed very thoroughly, incubated on a thermal cycler at 40°C for 20 h followed by 65°C for 10 min, and then held at 4°C. After MDA, the plate was purified by adding 20 μL Kapa pure beads (Kapa Biosystems, Wilmington, MA, USA) according to the manufacturer’s recommendations, including incubation for 7 min, washing twice with 80% ethanol, drying, and elution in 40 μL of 5 mM Tris-HCl (pH 8.0) at 37°C for 5 min. The supernatant containing purified DNA was then transferred to a clean plate.

### Near-full-length proviral amplification and sequencing.

Purified DNAs from MDA reactions were screened for proviruses using NFL PCR by diluting 2 μL of DNA with 8 μL of 5 mM Tris-HCl (pH 8.0). Diluted MDA DNA (2 μL) was then used for NFL PCR using the Bioline (London, UK) 2× Ranger polymerase PCR kit in 10-μL reactions with 400 nM HIV-1 NFL primers (outer primers for PCR1 and inner primers for nested PCR [See Table S2 in supplemental materials]) and the following PCR program for both rounds of PCR: (i) 95°C for 3 min, (ii) 98°C for 10 s, (iii) 57°C for 10 min, (iv) repetition of the second and third steps 29 times, (v) 57°C for 10 min, and (vi) a 10°C hold. The first-round PCR product was diluted 1:9 in 80 μL of 5 mM Tris-HCl (pH 8.0), and 2 μL of the diluted product was used to seed the nested PCR mixtures, after which the nested NFL PCR product was diluted with 40 μL of 5 mM Tris-HCl (pH 8.0). Positive NFL reactions were determined by a gel red plate readout using 5 μL of the diluted nested PCR product with 15 μL of 1× gel red nucleic acid stain (Biotium, Fremont, CA, USA), visualized at 302 nm on a transilluminator. The contents of positive gel red plate readout wells were analyzed for purity and size using 0.8% sodium borate agarose gel electrophoresis for 20 min at 250 V. Wells with single-band amplicons were purified by adding 36 μL of Kapa pure beads (Kapa Biosystems) according to the recommendations of the manufacturer. The purified amplicon was transferred to a clean sample well and sequenced as described below.

### Integration site library construction from NFL-positive MDA reactions.

MDA DNA selected for integration site analysis was quantified with Quant-iT dsDNA PicoGreen reagent (Biotium, Fremont, CA, USA), and an aliquot was normalized to 50 ng/μL in 5 mM Tris-HCl (pH 8.0) to a final volume of 25 μL. Quadruple restriction digestion was performed simultaneously on 1 μg of MDA DNA under the conditions recommended by the manufacturer (New England BioLabs, Ipswich, MA, USA), using CutSmart buffer and 0.45 U/μL of EcoRI-HF, BstZ17I-HF, NcoI-HF, and BamHI-HF, in a final volume of 25 μL. Digested DNA was then diluted with 25 μL of 5 mM Tris-HCl (pH 8.0), purified by adding 30 μL Kapa pure bead (Kapa Biosystems) as described above, and eluted in 25 μL of 5 mM Tris-HCl (pH 8.0). Samples were then end repaired by the addition of 25 μL of cold end repair master mix (final reaction mixture concentrations of 30 mM Tris-HCl [pH 8.0], 50 mM NaCl, 10 mM MgCl_2_, 5 mM DTT, 100 μM dNTPs, 100 ng/μL BSA, 30 μM NAD^+^, 0.025% Triton X-100, 0.05 U/μL Klenow large fragment, 0.03 U/μL T4 DNA polymerase, and 0.2 U/μL Escherichia coli ligase) and incubated at 20°C for 30 min, followed by a hold at 4°C. Samples were purified after end repair by adding 50 μL Kapa pure beads (Kapa Biosystems), eluted in 30 μL of 5 mM Tris-HCl (pH 8.0), and transferred to a new sample well. Following purification, samples were dA tailed by adding 20 μL of dA-tailing master mix (final reaction mixture concentrations of 15 mM Tris-HCl [pH 8.0], 50 mM NaCl, 10 mM MgCl_2_, 5 mM DTT, 100 μM dATP, 100 ng/μL BSA, 0.025% Triton X-100, and 0.25 U/μL Klenow Exo^−^) and incubated at 37°C for 30 min, followed by a 4°C hold. After dA tailing, 6.2 μL of 50 μM preannealed nullomer adapter was added to each sample, followed by the addition of 18.8 μL of ligation master mix (final reaction mixture concentrations, including dA tail mix, of 10 mM MgCl_2_, 10 mM DTT, 1.33 mM ATP, 7.5% polyethylene glycol 6000 [PEG 6000], and 40 U/μL T4 DNA ligase), and the samples were incubated for 4 h at 20°C and then either for 4 h at 16°C or overnight at 16°C. After ligation, samples were purified by adding 45 μL Kapa pure beads (Kapa Biosystems) to the plate, mixed, and incubated for 5 min. The supernatant was removed and discarded, and the beads were washed twice with 200 μL of 80% ethanol for ≥30 s each. After washing, the ethanol was removed, and the beads were dried for 3 min, followed by resuspension in 50 μL of 5 mM Tris-HCl (pH 8.0) and incubation at room temperature for 5 min. Purified DNA was transferred to a new sample well.

### Integration site PCR amplification from nullomer-ligated MDA DNA.

Nullomer-ligated DNA was diluted 1:5 with 5 mM Tris-HCl (pH 8.0), and 2 μL was used to seed PCR mixtures in quadruplicate. Two rounds of PCR were performed in a 10-μL reaction volume (2 μL nullomer-ligated DNA and 8 μL master mix) (final reaction mixture concentrations of 1× SuperFi buffer, 200 μM dNTPs [New England BioLabs], 250 nM adapter-specific primer, 250 nM HIV-specific primer, 84 μg/mL bovine thrombin [Sigma-Aldrich, St. Louis, MO, USA], 0.9 M PCR-grade betaine [Sigma-Aldrich], 8 ng/μL Extreme Thermostable Single Stranded Binding Protein (ET SSB) [McLab, San Francisco, CA, USA], and 0.02 U/μL Platinum SuperFi DNA polymerase [Thermo Fisher Scientific, Waltham, MA, USA]), and PCR was performed under the following conditions: (i) 95°C for 2 min, (ii) 98°C for 10 s, (iii) 66.5°C for 2 min, (iv) repetition of the second and third steps 39 times, (v) 66.5°C for 4 min, and (vi) a 10°C hold (See Table S2 in supplemental materials). The first-round PCR product was diluted with 80 μL of 5 mM Tris-HCl (pH 8.0), and 2 μL from each reaction mixture was used to seed the nested PCR mixtures. After nested PCR, the plate was diluted with 40 μL of 5 mM Tris-HCl (pH 8.0) and used for EvaGreen U5 amplicon qPCR detection.

### Detection of successful integration site amplification by EvaGreen U5 qPCR.

Integration site PCR mixtures were screened for U5 enrichment using EvaGreen qPCR (Biotium). Integration site nested PCR wells were diluted 1:1,200 in 5 mM Tris-HCl (pH 8.0), and 3 μL of the diluted PCR product was added to 7 μL of EvaGreen qPCR master mix (final reaction mixture concentrations of 1× ThermoPol buffer, 1.5 mM MgSO_4_, 0.4 mM dNTPs, 250 nM U5 forward primer, 250 nM U5 reverse primer, 1× EvaGreen dye, 50 nM ROX passive dye, 100 ng/μL BSA, and 0.025 U/μL HotStart *Taq*) and amplified using the following PCR program: (i) 95°C for 3 min, (ii) 95°C for 15 s, (iii) 57°C for 20 s, (iv) 72°C for 20 s (read fluorescence), (v) repetition of the second through fourth steps 29 times, and (vi) melt curve. Reaction mixtures producing a threshold cycle (*C_T_*) value of <25 were selected for 0.8% sodium borate agarose gel electrophoresis (data not shown).

### Purification and sequencing of integration site amplicons.

Amplicons of >772 bp (688 nt for the 5′ LTR and *gag* leader, 64 nt for the nullomer adapter, and a minimum of 20 nt of flanking host sequence) were purified by adding 45 μL Kapa pure beads (Kapa Biosystems) as described above. Purified DNA was eluted in 40 μL of 5 mM Tris-HCl (pH 8.0) and transferred to a new sample well. Integration site amplicons were then quantified using Quant-iT dsDNA PicoGreen reagent (Biotium) and sequenced using dideoxy and/or Illumina MiSeq methodologies (described below).

### Illumina MiSeq sequencing of NFL and integration site amplicons.

Amplicons underwent Illumina library construction using an in-house TruSeq dual-index-style workflow (Illumina, San Diego, CA, USA) or using the plexWell Plus 24 kit (seqWell, Beverly, MA, USA) according to the manufacturer’s instructions. Sequencing was performed using 500-cycle MiSeq nano v2 kits (Illumina) with paired-end 250-bp read lengths.

### Assembly of Illumina MiSeq sequencing data.

FASTQ files were aligned and consensus sequences were assembled/extracted using the CLC Genomics Workbench 12 *de novo* assembler (Qiagen, Germantown, MD, USA) (default settings except for a word size of 35) and/or the CLC Genomics Workbench 12 resequencing reference-guided mapping tool using donor-specific HIV-1 reference sequences (default settings, with the consensus extracted using a coverage filter of 40 reads). The consensus sequences generated were verified by BWA-MEM alignment of the raw FASTQ files using the generated consensus sequence as the reference (36). The quality of CLC assemblies was visually inspected, any identified errors were manually corrected, and corrections were reconfirmed using BWA-MEM alignment against the new consensus sequence. Consensus sequences from the IS and NFL amplicons were aligned by overlapping *gag* leader sequences using Sequencher v5.4.6 (Gene Codes, Ann Arbor, MI, USA) to generate the final integrated proviral consensus sequence.

### Quality assessment of proviral sequence data.

The final proviral consensus sequences were assessed for quality, inferred intactness, and LTR symmetry using the Proviral Sequence Annotation & Intactness Tool (ProSeq-IT), an online tool of the HIV-1 Proviral database of the National Cancer Institute (29). The tool was used to determine the inferred intactness of NFL sequences and compare the sequence identities between the two LTRs of each provirus. Samples with more than 3 mismatches between the LTRs or that had FASTQ files containing mixed bases at a given alignment position were deemed proviral mixtures and were excluded.

### Identification of the 5′ integration site from sequencing data.

IS amplicon consensus sequences were aligned against a consensus B HIV-1 LTR, and the flanking host sequence immediately upstream of the HIV-1 5′-LTR sequence was analyzed using the UCSC BLAT tool (37). Genomic locations with the highest sequence concordance were used for integration site location calling against the hg38 consensus genome. Analyses of data and the generation of Circos plots illustrating the distribution of ISs throughout the human genome were performed using Viral Genome Annotation System (Vgas) software (38).

### Analysis of proviral sequence data.

Proviral sequences were aligned in CLC Genomics Workbench using MUSCLE after trimming off flanking host sequences. Phylogenetic trees for each donor were constructed in MEGA6 by aligning the first 2 kb of each provirus, retaining gaps, and performing a neighbor-joining test of phylogeny using the bootstrap method (*n* = 1,000 replicates) (39). The overall mean pairwise distances of the proviruses (excluding hypermutants) for each donor were also calculated with MEGA6 using the bootstrap method (*n* = 1,000 replicates). Proviral sequence intactness was assessed with the ProSeq-IT pipeline (29). The clonality of proviruses was assessed and defined by those proviruses with identical integration sites and identical proviral sequences within 1 mismatch.

### Proviral sequence validation using host-to-full-length-provirus-to-host (HFH) nested PCR.

Integrated proviral sequences were validated by integration-site-specific nested PCR from unamplified PBMC gDNA as previously described (15). Integration-site- and provirus-specific PCR primers were designed using the IS and NFL amplicon sequences. gDNA was diluted 1:15 in 5 mM Tris-HCl (pH 8.0), and 2 μL was seeded into all wells of a PCR plate containing 8 μL of either Platinum SuperFI DNA polymerase master mix (final reaction mixture concentrations of 1× SuperFI buffer, 0.2 mM dNTPs, 250 nM IS-specific primer, 250 nM HIV-1 primer, and 0.02 U/μL Platinum SuperFI DNA polymerase [Thermo Fisher Scientific]) or 2× Ranger master mix (final reaction mixture concentrations of 1× Ranger mix, 400 nM IS-specific primer, and 400 nM HIV-1 primer [Bioline]). Thermal cycling parameters were dependent upon each provirus and the host sequence flanking the provirus (data not shown). Amplicons generated were sequenced by Illumina MiSeq (Illumina) and assembled by the CLC Workbench 12 *de novo* assembler (Qiagen) as described above.

### Data availability.

Proviral sequences and integration sites will be submitted to the publicly accessible Proviral Sequence Database (PSD) and the Retrovirus Integration Database (RID) (https://rid.ncifcrf.gov/) at the U.S. Department of Health and Human Services, National Institutes of Health, National Cancer Institute. Sequences submitted to the GenBank database can be found under accession numbers MZ868394 to MZ868495. Raw sequence files submitted to the Sequence Read Archive (SRA) can be found under accession number PRJNA742825.
